# Rotundic Acid Induces DNA Damage and Cell Death in Hepatocellular Carcinoma Through AKT/mTOR and MAPK Pathways

**DOI:** 10.3389/fonc.2019.00545

**Published:** 2019-06-26

**Authors:** Gaurab Roy, Su Guan, Hexiang Liu, Lei Zhang

**Affiliations:** ^1^School of Biology and Biological Engineering, South China University of Technology, Guangzhou, China; ^2^Guangdong Provincial Engineering and Technological Centre for Biopharmaceuticals, South China University of Technology, Guangzhou, China

**Keywords:** rotundic acid, hepatocellular carcinoma, DNA damage, apoptosis, anti-angiogenesis

## Abstract

Hepatocellular carcinoma (HCC) is the fourth largest cause of cancer-related deaths worldwide with limited therapeutic interventions. Renewed interest in natural products as drug leads has resulted in a paradigm shift toward the rapid screening of medicinal plants for the discovery of new chemical entities. Rotundic acid (RA), a plant-derived triterpenoid, has been anecdotally reported to possess anti-inflammatory and cardio-protective abilities. The present study highlights the anti-cancer efficacy of RA on HCC *in vitro* and *in vivo*. The inhibitory effects of RA on HCC cell viability was determined by MTT. Soft agar colony formation and clonogenic assays also showed that RA inhibited HCC cell proliferation. Flow cytometry, confocal, and western blot results further indicated that RA induced cell cycle arrest, DNA damage, and apoptosis by modulating the AKT/mTOR and MAPK pathways. Besides the suppression of migration and invasion, tube formation and VEGF-ELISA revealed the anti-angiogenic abilities of RA on HCC. Moreover, RA also inhibited tumor growth in a HepG2 xenograft mouse model. To our best knowledge, this is the first extensive study of the anticancer activity of RA on HCC. The results demonstrate that RA could be a potential drug candidate for HCC treatment.

## Highlights

- Rotundic acid (RA) exhibits anticancer activity against hepatocellular carcinoma (HCC).
- RA inhibits tumor growth, progression, and angiogenesis in HCC cells *in vitro*.
- RA abrogates tumor growth in Balb/c nude mice.
- RA can act as a potential lead compound for HCC drug development.

## Introduction

Hepatocellular carcinoma (HCC) is the fourth largest cause of cancer-related deaths worldwide. The global burden of HCC is on the rise with Asia alone contributing to more than 70% of the total HCC cases worldwide (1). Despite recent advances in diagnosis and treatment; poor prognosis, high recurrence, and rapid progression make HCC a highly lethal disease (2). Neovascularization plays a crucial role in HCC development, and consequently, malignancies of the liver are usually found to be hyper-vascular in nature. Tumor progression, angiogenesis, and metastases are the major causes underlying HCC related deaths. As a result, there has been a growing interest focussed toward the role of new blood vessel formation in the pathogenesis of HCC (3, 4). Sorafenib, a multiple kinase inhibitor, has emerged as a standard treatment for advanced HCC (5). More recently, the FDA approved Regorafenib for HCC treatment (6, 7). Regorafenib, like its predecessor, inhibits receptor tyrosine kinases, their downstream mediators and angiogenesis (8). Although these targeted agents improve survival; limited therapeutic benefits and absence of durable responses have led to the suboptimal use of anti-angiogenic therapies (9). Inefficient therapeutic interventions urge for the rapid development of new therapies for this aggressive disease with the aim of improving treatment response and minimizing toxicity for patients with HCC (10).

Natural products serve as excellent sources for the development of novel and contemporary medications for disease treatment. More than 70% of the FDA approved drugs between 1981 and 2014 are either natural products or their derivatives (11). Natural products represent the richest source of novel molecular scaffolds, and hence over the last few decades, there has been a renewed and increasing interest in developing natural products as drug candidates (12). Both pre-clinical and clinical trials have successfully demonstrated the importance of natural products against HCC and other diseases (13, 14).

The barks of *I. rotunda*, commonly known as “*Jiu Bi Yin*g” in China, is been used extensively as traditional Chinese medicine (TCM) for the treatment of common cold and fever, urinary tract infection, bone pain, and cardiovascular diseases (15). The crude extract of this plant has been reported to possess diverse bioactivities including protecting the cardiovascular system, anti-inflammation, anti-bacterial, anti-oxidation, and has been officially recorded in the Chinese Pharmacopeia since 2010 (16, 17). Triterpenoids are the major bioactive compounds obtained from the bark and leaves of *I. rotunda* along with diterpenoids, lignans, phenolic acids, and steroids (18). Rotundic Acid (RA), a naturally occurring triterpenoid obtained from *I. rotunda*, has been reported to possess anti-inflammatory and cardio-protective abilities (19). RA and its derivatives have also been reported to induce cell death by apoptosis in various cancers (20). However, a comprehensive analysis of the effects of RA on HCC and its mechanisms of action are yet to be delineated. In the present study, we have systematically investigated the anti-cancer efficacy of RA under *in vitro* and *in vivo* conditions. We employed MTT, colony formation, cell migration, and invasion assays to determine the inhibitory effects of RA on HCC cells (HepG2 and SMMC-7721). The inhibitory mechanisms were determined by cell cycle analysis, DNA damage assay, Annexin V-FITC/PI staining, western blot, tube formation assay, and VEGF-ELISA. Moreover, the Balb/c nude xenograft mouse model was also utilized to evaluate the restrictive effects of RA on HCC *in vivo*.

## Materials and Methods

### Cell Culture

Human HCC cell lines (HepG2, SMMC-7721) and human umbilical vein endothelial cells (HUVEC) were generously provided by the School of Pharmaceutical Sciences, Sun Yat-sen University (Guangzhou, China) and were grown in DMEM, RPMI-1640, and endothelial cell medium (ScienCell Research Laboratories, Carlsbad, CA), respectively. LO2 cells were purchased from Gaining Biological (Shanghai, China) and cultured in DMEM. Culture mediums for the hepatocytes were supplemented with 10% FBS, 1% penicillin and streptomycin solution. The cells were maintained in a humidified atmosphere of 5% CO_2_ at 37°C. All cell culture reagents were purchased from Life Technologies, Inc. (Gaithersburg, MD) unless mentioned specifically.

### Cell Viability and Proliferation

The viability of cells was determined by MTT assay as reported earlier (21). 1 × 10^4^ HCC, endothelial (HUVEC), and normal hepatic cells (LO2 cells) were seeded per well in 96 well plates, respectively. After 24 h incubation in growth factor supplemented medium, the cells were treated in triplicate with various concentrations of RA for 24, 48, and 72 h, respectively. MTT (Aladdin, Shanghai, China) was added and readings were taken at 570 nm using the SpectraMax 190 microplate reader (Molecular Devices, Sunnyvale, CA). Furthermore, the long-term effects of RA on HCC cell proliferation was established by the clonogenic assay as described previously (22). HCC cells were trypsinized following 24 h incubation with various concentrations of RA. Cells were counted and 10^3^ control and RA-treated HCC cells were grown in a controlled humidified environment for another 10 days, with media change every 2–3 days. Cells were then fixed using 4% paraformaldehyde (PFA) solution and stained with 0.05% crystal violet (Amersco, China) solution. Images were taken using the FluorChem M imaging system (proteinsimple, San Jose, CA). Experiments were repeated thrice.

### Anchorage-Independent Soft Agar Colony Formation Assay

5 × 10^3^ RA-treated HCC cells were mixed with 0.3% agarose and were plated over a 0.6% agarose layer (Biowest, Spain) as mentioned previously (23). The medium was renewed twice weekly. After 21–30 days, the cells were fixed using 4% PFA, washed, and further stained with 0.05% crystal violet solution. The colonies were photographed at 40× magnification using a camera fitted inverted microscope. Cell colonies with more than 25 cells were counted manually in five randomly selected magnification fields and the data are represented as % of control. At least three independent experiments were performed.

### Migration Assays

Wound migration assay was carried out as described previously (24). 4 × 10^4^ HepG2 and SMMC-7721 cells were seeded in 24 well plates and allowed to grow till confluence, respectively. The confluent monolayers of the HCC cells were gently scratched using 200 μl pipette tips and the cells were treated with the indicated concentrations of RA. Wound images were taken using a camera fitted inverted microscope at 0 and 16–24 h, respectively. Area migrated by the cells (A) was calculated as A = (Wound Area at 0 h–Wound Area at 16–24 h) and are expressed as % wound closure. A minimum of three independent repeats was carried out.

Boyden chambers were utilized to examine the influence of RA on the motility of endothelial cells. Briefly, 3 × 10^4^ RA-treated HUVEC's were allowed to migrate through the Boyden chambers under the influence of a chemo-attractant (10% FBS). The migrated cells were stained with 0.05% crystal violet solution and the images were obtained using an inverted microscope fitted with a camera adapter. The experiment was repeated thrice.

### Invasion Assay

Cell invasion assay was performed using Matrigel (Corning, USA) coated transwell inserts (25). The transwells were coated with 100 μL of 250 μg/mL Matrigel solution and were left undisturbed for 2 h at 37°C. RA-treated HCC cells were trypsinized and 3 × 10^4^ cells were plated on the Matrigel-coated Boyden chambers. Following 24 h incubation in a controlled environment, the cells were fixed with 4% PFA for 20 min, washed with PBS, and then stained with 0.05% crystal violet in 20% methanol for another 30 min. The transwells were washed thrice using PBS and cells in the upper chambers were removed by gently dabbing them with cotton swabs. Images were acquired at 100–200× magnifications and the number of invading cells in five random fields of view was counted manually. Data are represented as % of control. Three independent repeats were carried out.

### Gelatin Zymography

Conditioned medium of RA-treated HCC cells was collected, dialyzed, and lyophilized. Ten microgram total protein was loaded per well and the experiment was carried out as reported earlier (26). The gel was washed twice with wash buffer (2.5% Triton X-100, 50 mM Tris-HCl, 5 mM CaCl_2_, 1 μM ZnCl_2_) and incubated in freshly prepared incubation buffer (1% Triton X-100, 50 mM Tris-HCl, 5 mM CaCl_2_, 1 μM ZnCl_2_) for 20 h at 37°C. The gel was then stained using 0.5% Coomassie brilliant blue in 50:40:10 water: methanol: acetic acid solution for 30 min followed by de-staining the gel with two washes of 50:40:10 water: methanol: acetic acid solution for 30 and 90 min, respectively. Gel images were taken using the FluorChem M imaging system and the band intensities were calculated by ImageJ software (NIH). The experiment was repeated thrice and the results are expressed as % of control.

### Cell Cycle Analysis

The cell cycle analysis of RA-treated HepG2 cells was performed as described previously (27). In brief, the control and RA treated HepG2 cells were fixed overnight in 70% ethanol and then incubated with PBS containing 1 mg/mL propidium iodide (Sigma-Aldrich) and 0.1 mg/mL RNase (Sigma-Aldrich) at room temperature in dark for 30 min. The fluorescence intensities of the DNA-bound propidium iodide in HepG2 cells were measured using a FACScan flow cytometer (BD Biosciences, San Jose, CA) and the results are indicative of the % of total cells in the G1, S, or G2 phase of the cell cycle. Three independent experiments were performed.

### DNA Damage

Nuclear damage and morphological changes induced by RA in HepG2 cells were detected by Hoechst 33342 staining (Sigma-Aldrich) (28). Briefly, RA-treated HepG2 cells were fixed with 4% PFA and the cell nucleus was stained using 5 μg/mL Hoechst 33342 for 15 min at room temperature. The unbounded dye was washed with PBS and the images were acquired using a fluorescence microscope. The number of the damaged and deformed nucleus was counted manually in five randomly selected magnification fields and is expressed as % of control. Three repeats were performed.

### Annexin V-FITC/PI Staining

Annexin V-FITC/PI staining kit was obtained from Beyotime biotechnology (Shanghai, China). HCC cells (4 × 10^5^ cells/well) were grown in 60 mm cell culture dishes for 24 h and used for Annexin V-FITC/PI experiments as mentioned earlier (29). Briefly, the RA treated HCC cells and the control cells were trypsinized, washed twice with PBS, and collected at a concentration of 5 × 10^5^ cells/ml in binding buffer. Cells were then incubated with fluorescein isothiocyanate (FITC)-labeled Annexin V (10 μg/mL) and PI (20 μg/mL) at 37°C for 20 min in the dark and analyzed with an Accuri™ C6 flow cytometer (BD Biosciences, USA) using excitation/emission wavelengths of 488/525 nm and 488/675 nm for Annexin V and PI, respectively. Apoptosis was determined by the sum of the percentage of cells present in the Annexin V +ve/PI –ve and Annexin V +ve/PI +ve quadrants.

### Tube Formation Assay

Human Umbilical Vein Endothelial Cells (2 × 10^4^) were re-suspended in RA-treated HepG2 cell conditioned medium. The cells were then plated in a 96 well plate coated with 10 mg/mL Matrigel (30). HUVEC cells grown in the conditioned medium (CM) of HepG2 cells without RA treatment acted as the control specimen. Images were taken after 6 h incubation using a Nikon microscope fitted with a camera adapter at 100× magnification. The number of tubes was counted manually in five random fields of view and the end-result is represented as % of control. The experiment was repeated thrice and in all experiments, primary HUVECs were used at passages 2–7.

### Immunoblotting

The following antibodies were purchased from Cell Signaling Technology (Danvers, USA): phospho-mTOR (Ser2448), mTOR, phospho-Akt (Ser473), Akt, phospho-p44/42 MAPK (Erk1/2) (Thr202/Tyr204), p44/42 MAPK (Erk1/2), phospho-p38 MAPK (Thr180/Tyr182), p38 MAPK, Bax, Bcl-2, PARP, cleaved caspase-3, GAPDH, and HRP conjugated secondary antibodies. Caspase-3, Ki-67, and CD-31 antibodies were acquired from ABclonal Technology, China. Protein samples were denatured and separated on an SDS-PAGE gel. After the gel run, the proteins were transferred on to a PVDF membrane by sandwiching the gel and the membrane between sponges and filter paper. Transfer was carried out at 225 mA for 150 min at 4°C. The PVDF membrane was then blocked using 5% BSA in TBS-T (0.1% Tween-20 in TBS) for 120 min. The membrane was further washed thrice with TBS-T and incubated overnight at 4°C with primary antibody. The primary antibody was removed and the membrane was again washed three times with TBS-T solution for 10 min each. The PVDF membrane was then incubated with secondary antibody for 90 min at room temperature and the chemiluminescence signals were visualized by FluorChem M imaging system. All primary and secondary antibodies were used at dilutions of 1:2,000 and 1:5,000, respectively. Experiments were repeated thrice.

### ELISA

Human VEGF ELISA kit (ARG80124) was purchased from Arigo bio-laboratories, Taiwan, ROC, and experiments were performed according to the manufacturer's instructions. The optical density of the test samples was plotted against a VEGF standard curve. Samples were analyzed in duplicates and three independent repeats were carried out.

### Tumor Xenograft Study

Male nude mice (Balb/c strain), 5 weeks old, were purchased from Hunan SJA Laboratory Animal Co., Ltd. (Changsha, China). Mice were housed under pathogen-free conditions and were allowed free access to food and water at their own will. The study protocol was approved by the Institutional Animal Care and Use Committee of South China University of Technology (Guangzhou, China, Ref: 20180009).

Mice xenograft model was used to investigate the anti-tumor effects of RA *in vivo*. The right flanks of the mice were injected subcutaneously with 5 × 10^6^ HepG2 cells. After the formation of palpable tumors, mice were randomly divided into two groups (3 mice per group) and were treated with intraperitoneal injections of HBSS and 50 mg/Kg RA every 2 days. Tumor dimensions and body weights were measured simultaneously. The tumor dimensions were measured using a digital vernier caliper and the tumor volumes were calculated as V = (width)^2^ × (length)/2. Mice were sacrificed after 60 days and the tumors were extracted, weighed, and photographed. The tumor growth inhibition (TGI) was assessed by the formula 1–(mean tumor volume of treatment group/mean tumor volume of the control group) (31). WB was further performed with the tumor tissue lysates as described in section Immunoblotting and the blots were developed using the western blot and chemi-imaging system FUSION Solo S (Vilber smart imaging, France).

### Statistical Analysis

Statistical analysis was performed using GraphPad Prism 7.0 (GraphPad, CA, USA). Data are represented as mean ± SD. Student's *t*-test was used to determine the statistical significance. Values of *p* ≤ 0.05 were considered significant.

## Results

### Effects of RA on the Growth of HCC and Endothelial Cells *in vitro*

**Figure 1:**
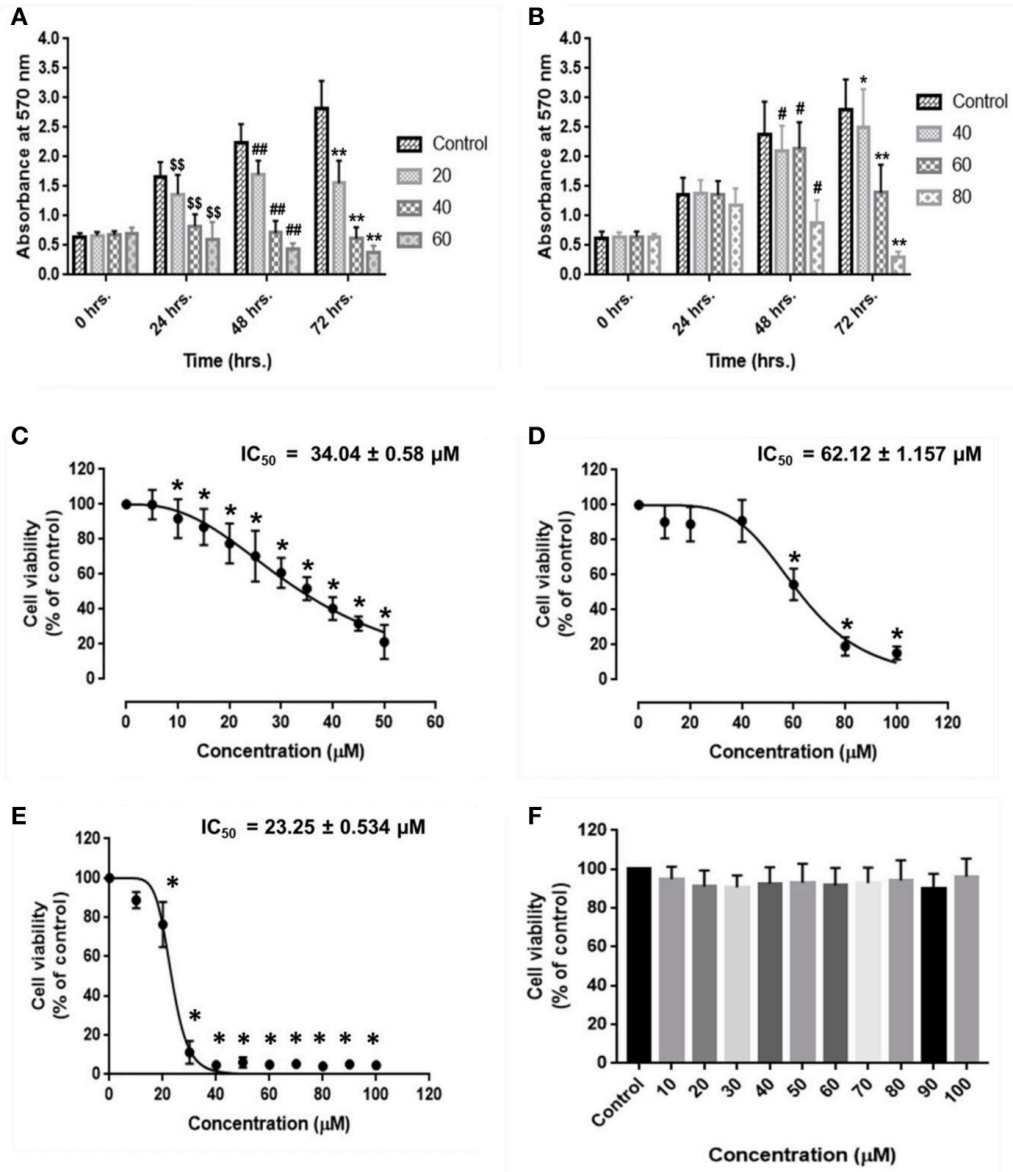
Inhibitory effects of rotundic acid (RA) on HCC and endothelial cell growth. MTT assays were performed to determine the effects of RA on HCC and endothelial cell growth. RA inhibited the cell viability and proliferation of **(A)** HepG2 cells, **(B)** SMMC-7721 cells in a time and dose-dependent manner. The dose survival curves of **(C)** HepG2 cells, **(D)** SMMC-7721 cells, **(E)** HUVEC's, and **(F)** LO2 cells after RA treatment. RA exhibited no significant toxicity on the normal hepatic LO2 cells. Data are represented as mean ± SD (*n* = 3; ^$$, ##^, **p ≤ 0.01 and ^#^, *p ≤ 0.05 vs. control).

HCC and endothelial cells were treated with RA in triplicate and MTT assay was performed. The results suggested that RA could inhibit the cell viability and proliferation of HepG2 (Figure 1A) and SMMC-7721 (Figure 1B) cells in a time and dose-dependent manner. RA also produced cell death of human umbilical vein endothelial cells (HUVEC) in a concentration-dependent manner. The IC_50_ values of RA obtained from the dose survival curves were found to be 34.04 ± 0.58 μM for HepG2 cells (Figure 1C), 62.12 ± 1.16 μM for SMMC-7721 cells (Figure 1D), and 23.25 ± 0.534 μM for HUVEC's (Figure 1E). HUVEC and HepG2 cells were found to be more sensitive to RA treatment as compared to the SMMC-7721 cells. Furthermore, to find out if the RA induced cell death was specific to cancer cells, we treated normal hepatocytes (LO2), in triplicate, with increasing concentrations of RA ranging from 10 to 100 μM. Fortunately, no significant cell death was observed in LO2 cells on RA treatment, indicating that RA had minimal toxic effects on normal hepatic cells as compared to HCC cells (Figure 1F).

**Figure 2:**
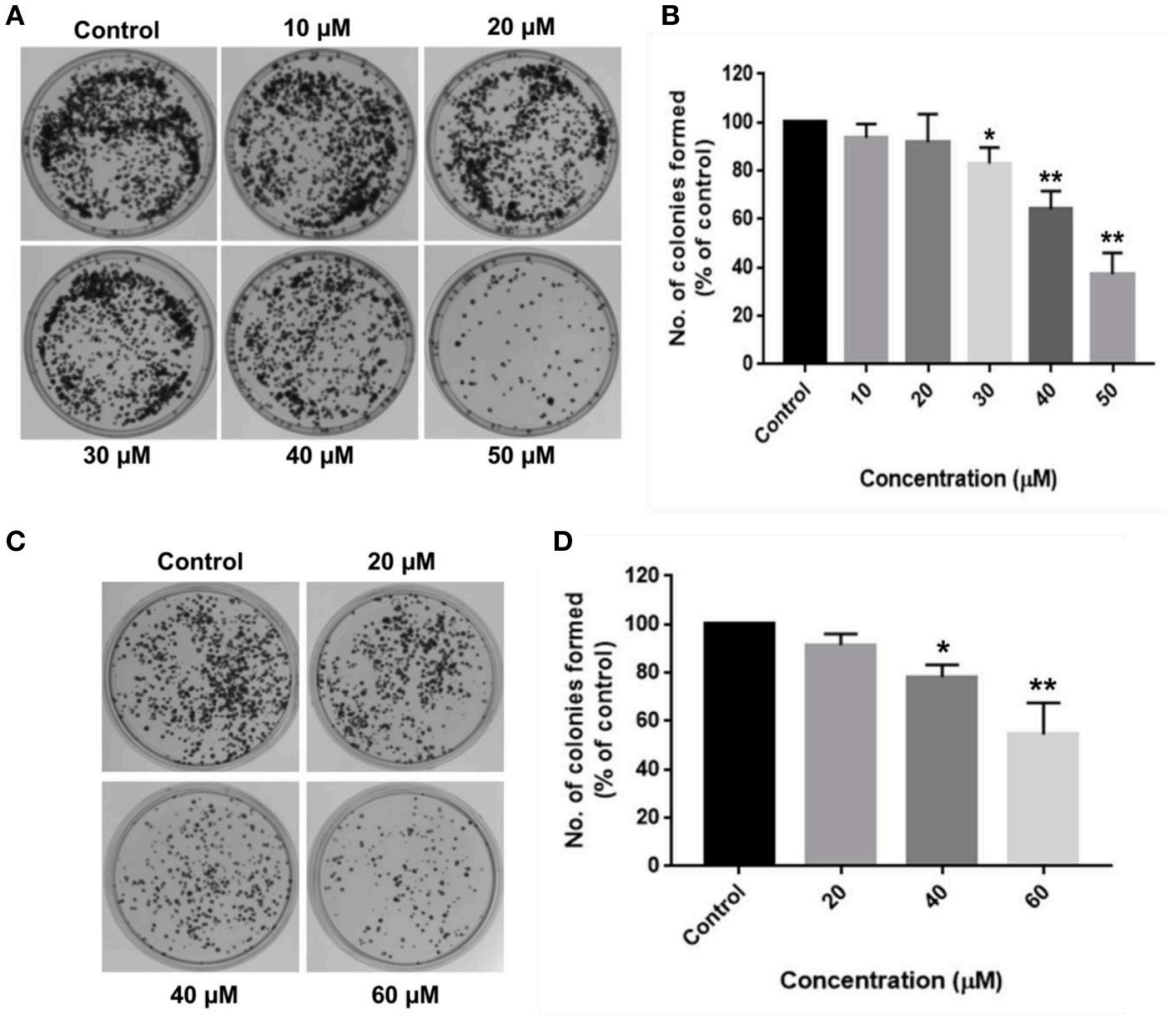
RA restricts the clonogenic properties of HCC cells *in vitro*. Clonogenic/Colony formation assay was performed to study the long-term effects of RA on the survival and proliferation of HCC cells. RA inhibited the colony forming ability of **(A,B)** HepG2 and **(C,D)** SMMC-7721 cells in a concentration-dependent manner. Data are represented as mean ± SD (*n* = 3, and **p ≤ 0.01, *p ≤ 0.05 vs. control).

Long-term inhibitory effects of RA on HCC cell growth were demonstrated by their inability to form colonies after RA treatment. HepG2 cells treated with 30 μM RA produced 20% lesser colonies when compared to the colonies formed by the untreated control cells. The inhibition was >60% in 50 μM RA treated HepG2 cells (Figures 2A,B). Similarly, more than 20% reduction in the number of SMMC-7721 cell colonies were observed on plating cells treated with 40 μM RA, which further escalated to almost 50% when the concentration of RA was increased to 60 μM (Figures 2C,D). The results exhibited a concentration-dependent reduction in the number of HepG2 and SMMC-7721 cell colonies, confirming the persistent effects of RA on HCC cell proliferation.

**Figure 3:**
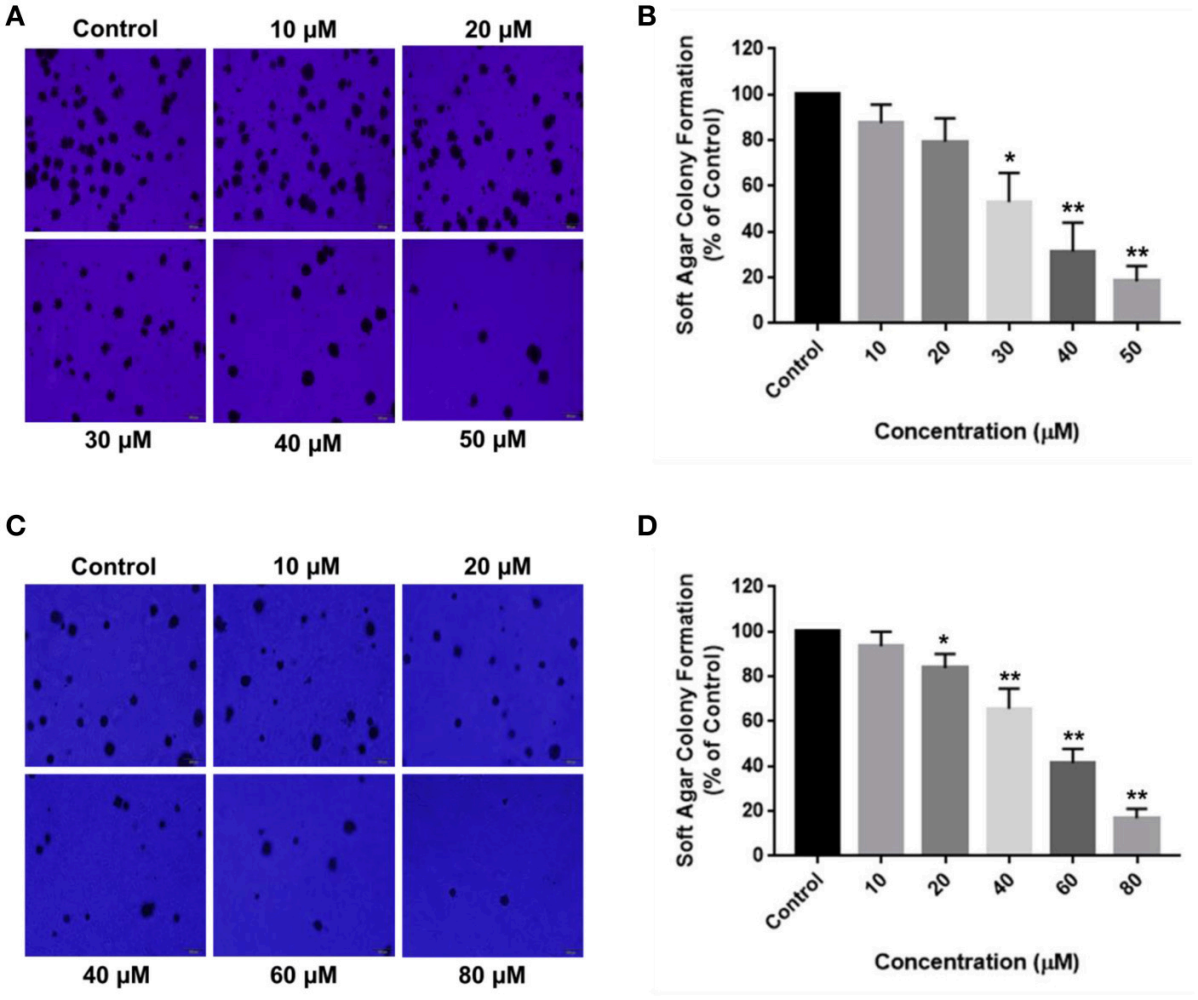
RA attenuates extracellular matrix-independent growth of HCC cells. RA treatment limited the anchorage-independent colony forming ability of **(A,B)** HepG2 and **(C,D)** SMMC-7721 cells in a dose-dependent manner. Data are represented as mean ± SD (*n* = 3, magnification = 40×, scale bar = 200 μm and **p ≤ 0.01, *p ≤ 0.05 vs. control).

Aberrant mutations in cancers enable cells to proliferate without attaching to the extracellular matrix (ECM). Soft agar colony formation assay is a well-established method to determine the tumorigenic potential of malignant cells by evaluating their ability to survive in an anchorage-independent manner. The inhibitory effects of RA on HCC cell growth were further validated by the anchorage-independent growth assay, where a marked difference was observed in the number of cell colonies in the soft agar. RA treatment resulted in a considerable decrease in the extracellular matrix-independent survival and proliferation of HepG2 and SMMC-7721 cells *in vitro*. HepG2 cells treated with 30 μM RA produced 40% lesser colonies on soft agar as compared to the untreated cells (Figures 3A,B). Higher concentrations of RA further inhibited the anchorage-independent colony forming ability of HepG2 cells. A similar reduction in the number of colonies formed by the RA treated SMMC-7721 cells were observed. Forty micromolar RA treatment resulted in 20–30% reduction in the soft agar colonies of SMMC-7721 cells *w.r.t* control and only 15–20% colonies *w.r.t* control were observed in the plates containing 80 μM RA treated SMMC-7721 cells (Figures 3C,D).

### RA Abrogates HCC Cell Migration, Invasion, and MMP-2/-9 Activities

**Figure 4:**
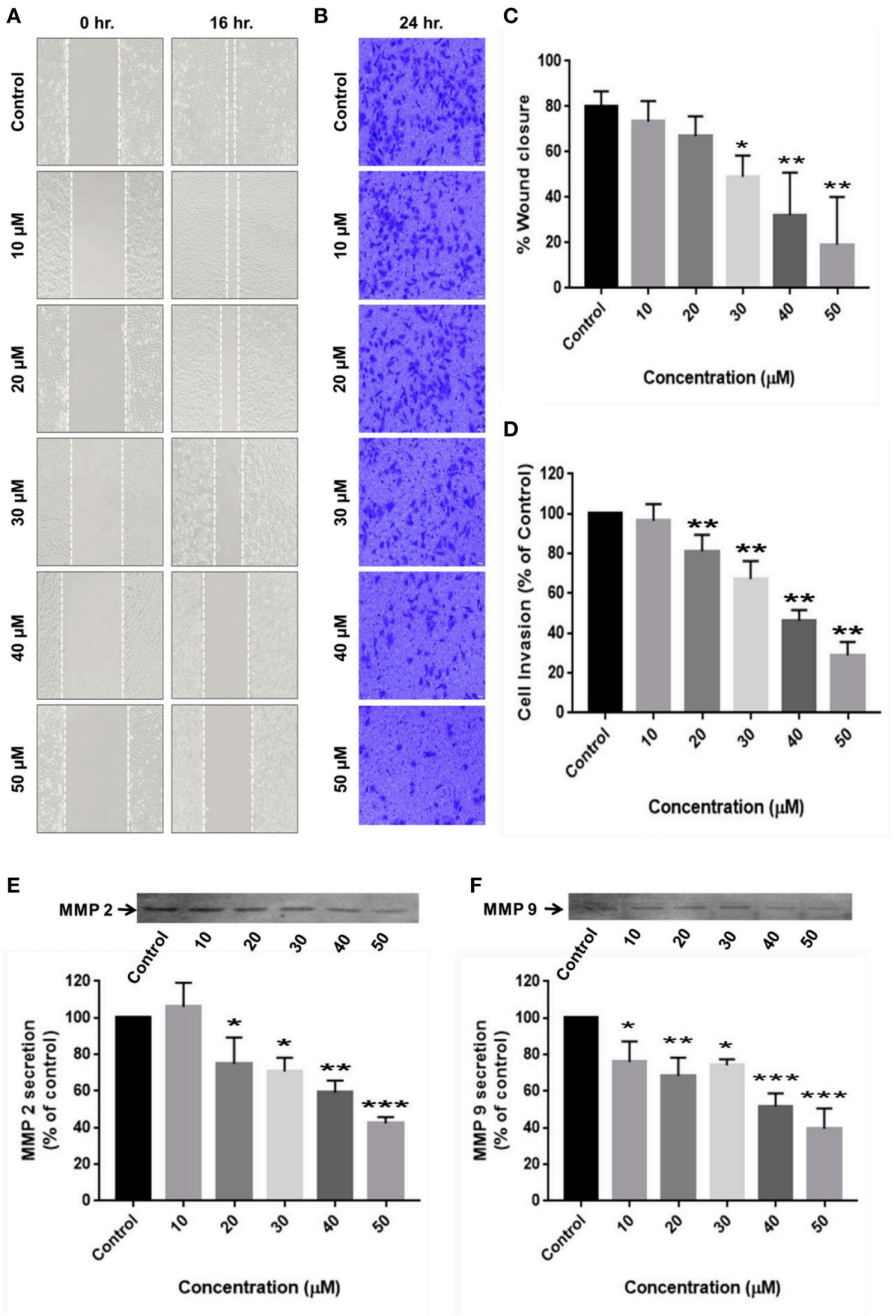
RA restricts the migration and invasion of HepG2 cells by inhibiting MMP-2/MMP-9 secretion. **(A,C)** RA inhibited the migration of HepG2 cells in a dose-dependent manner. **(B,D)** RA treatment weakened the ability of HepG2 cells to invade through the basement membrane in a concentration-dependent manner. RA restricted the secretion of matrix metalloproteinases **(E)** MMP-2 and **(F)** MMP-9 from HepG2 cells in a concentration-dependent manner. Data are expressed as mean ± SD. Images for migration and invasion assays were taken at 100 and 200× magnifications and the scale bars are 50 and 20 μm, respectively (*n* = 3 or more; ***p ≤ 0.001, **p ≤ 0.01, *p ≤ 0.05 vs. control).

Cell migration is indispensable for cancer cell invasion and metastasis. Wound healing and matrigel-coated transwell assays were performed to determine the ability of RA to curb cell motility and invasiveness of HCC cells. The results revealed that RA treatment successfully attenuated the wound migration (Figures 4A,C) and invasion (Figures 4B,D) of HepG2 cells in a concentration-dependent manner.

For cancer cells to metastasize to distant sites, they need to degrade and invade through the basement membrane. Matrix metalloproteinases (MMP's) enables tumor cells to disintegrate the extracellular matrix and enter the blood or lymphatic vessels through which they are transported to distant target organs and establish secondary tumors. Zymography was consequently performed to determine the reason underlying the anti-migration and anti-invasion effects of RA on HepG2 cells. The results exhibited a dose-dependent reduction in the secretion of matrix metalloproteinases (MMP-2 and MMP-9) from HepG2 cells upon RA treatment (Figures 4E,F).

**Figure 5:**
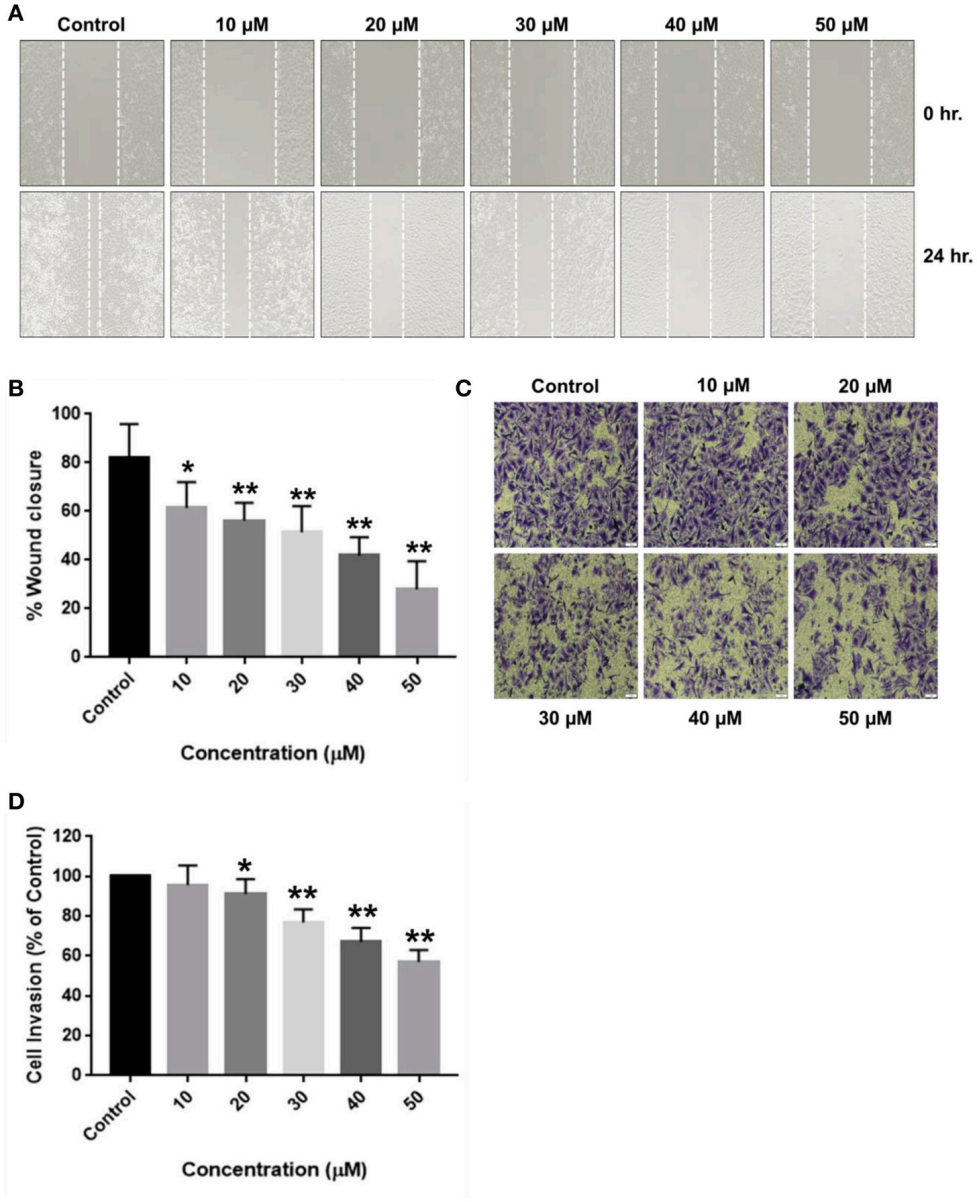
Inhibitory effects of RA on the migration and invasion of SMMC-7721 cells. SMMC-7721 cells were treated with various concentrations of RA and subjected to migration and invasion assays. RA treatment prevented the **(A,B)** wound closure and **(C,D)** extracellular matrix invasion of SMMC-7721 cells in a concentration-dependent manner (*n* = 3 or more; **p ≤ 0.01, *p ≤ 0.05 vs. control).

In a similar fashion, RA also restricted the migration (Figures 5A,B) and invasion (Figures 5C,D) of SMMC-7721 cells in a concentration-dependent manner. RA did not produce considerable decrease in the MMP secretion of SMMC-7721 cells at the indicated doses but, significant effects were observed at higher concentrations (Supplementary Figure S1). Our results demonstrate that rotundic acid has a promising role in the prevention of hepatocellular carcinoma tumor metastasis.

### RA Inhibits Cell Cycle, Causes DNA Damage, and Triggers Apoptosis in HepG2 Cells

**Figure 6:**
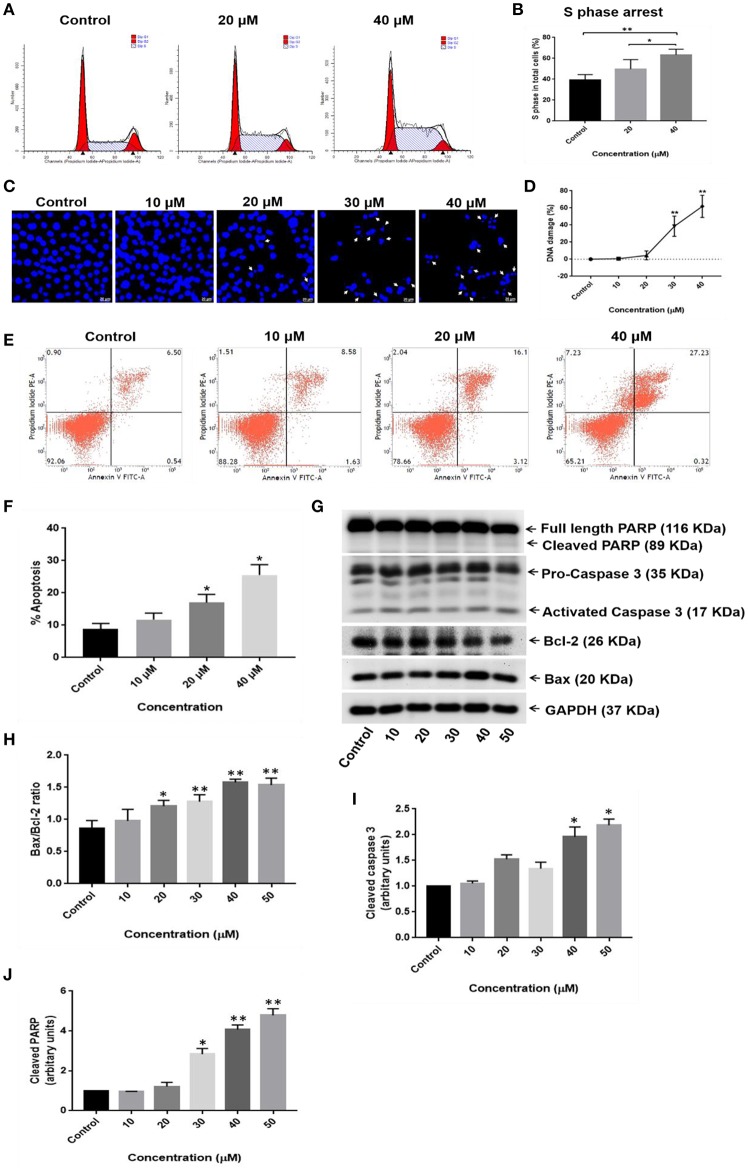
RA induces S-phase cell cycle arrest and apoptosis in HepG2 hepatocellular carcinoma cells. **(A,B)** RA treatment resulted in S-phase cell cycle arrest in HepG2 cells. **(C,D)** Morphological changes in the cell nucleus and nuclear fragmentation were observed upon RA treatment. **(E,F)** The pro-apoptotic effects of RA on HepG2 cells were determined by Annexin V-FITC/PI staining. **(G)** Western Blot analysis of apoptosis-related proteins like PARP, caspase-3, Bax, and Bcl2 indicated that RA induced apoptosis in HepG2 cells in a concentration-dependent manner. Densitometric analysis of **(H)** Bax/Bcl2 ratio, **(I)** cleaved caspase 3, and **(J)** cleaved PARP obtained in WB. Confocal images were taken at 400× magnification and the scale bar is 20 μm (*n* = 3; **p ≤ 0.01, *p ≤ 0.05 vs. control).

Cell cycle analysis was carried out to investigate the effects of RA on the cell cycle progression in HepG2 cells. RA treatment resulted in an increased accumulation of HepG2 cells in S-phase of the cell cycle (Figures 6A,B; Supplementary Figure S2A). Apoptosis is one of the major causes of cancer cell death and is accompanied by changes in the cellular morphology, nuclear degradation along with altered protein expressions. Therefore, nuclear staining was performed to check for the presence of the damaged nuclei and to determine whether apoptosis was involved in RA-mediated death of HepG2 cells. It was found that RA treatment led to nuclear damage and DNA fragmentation in HepG2 cells (Figures 6C,D; Supplementary Figure S2D). To further confirm that RA treatment triggered apoptosis in HepG2 cells, we performed Annexin V—FITC/PI staining of RA treated HepG2 cells and also determined the expression levels of the pro-apoptotic protein (Bax), anti-apoptotic protein (Bcl2), caspase-3, and PARP using western blot. Annexin V – FITC/PI staining indicated a concentration-dependent increase in the apoptotic cell population of HepG2 cells (Figures 6E,F). The WB results displayed a dose-dependent reduction in Bcl2 expression along with PARP cleavage and increased expressions of Bax, activated caspase-3 in RA-treated HepG2 cells (Figures 6G–J). Similarly, RA treatment also triggered apoptosis in SMMC-7721 cells (Supplementary Figures S2B,C). These results indicated that S-phase cell cycle arrest and apoptosis contributed to the RA-induced HCC cell death.

### RA Inhibits Angiogenesis *in vitro*

**Figure 7:**
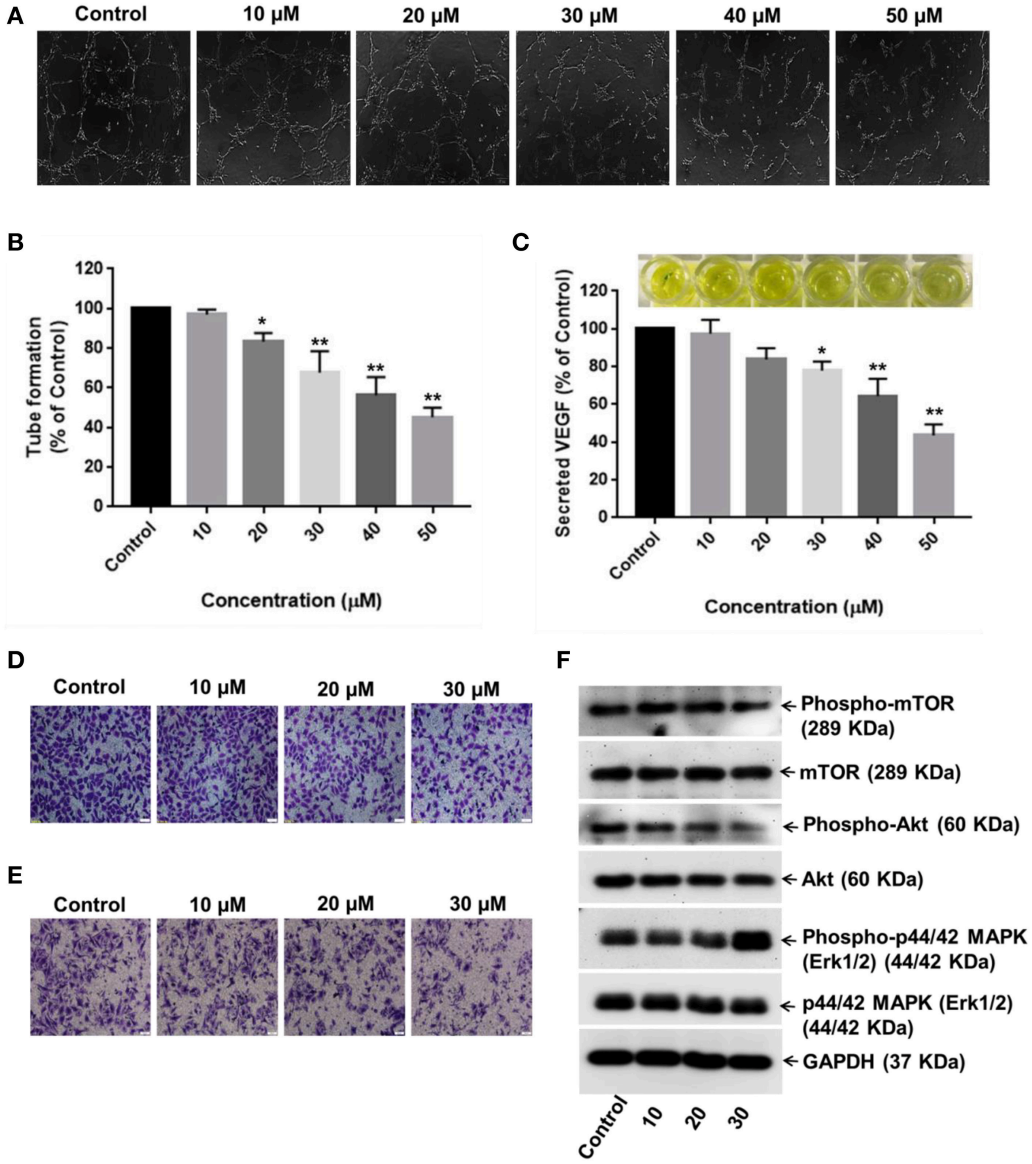
RA inhibits VEGF secretion and endothelial cell-mediated angiogenesis in HCC. **(A,B)** HUVECs grown in the conditioned medium of RA-treated HepG2 cells produced shortened and severely broken 3D-tubular networks on the basement membrane matrix in a dose-dependent manner. **(C)** RA treatment abated VEGF release from HepG2 cells. Moreover, RA treatment also impeded the **(D)** migration and **(E)** invasion of HUVEC cells in a concentration-dependent manner. **(F)** RA modulated the expressions of AKT/mTOR and MAPK pathway molecules in the endothelial cells (*n* = 3 or more, 100× magnification, scale bar = 50 μm, **p ≤ 0.01 and *p ≤ 0.05 vs. control).

Neovascularization and angiogenesis play important roles in HCC growth and progression. To determine whether RA inhibited endothelial cell-mediated angiogenesis in HCC, the effects of RA on HUVEC tube formation were examined. The anti-angiogenic ability of RA was revealed by the inability of HUVECs to form 3D-tubular structures on the basement membrane matrix when incubated with the conditioned medium (CM) of RA-treated HepG2 cells as compared to the HUVECs grown in the CM of untreated HepG2 cells (Figures 7A,B). The above outcome was further supported by the reduced VEGF (a highly specific mitogen for endothelial cells and a known angiogenesis inducer) concentrations in RA-treated HepG2 cell culture supernatants *w.r.t*. the untreated control cells (Figure 7C). Endothelial tube formation assay together with VEGF-ELISA highlighted the anti-angiogenic properties of RA in hepatocellular carcinoma. It was also shown that RA inhibited the transwell migration (Figure 7D) and invasion (Figure 7E) of HUVECs in a dose-dependent manner. The anti-angiogenic activities of RA could be attributed to its ability to attenuate VEGF secretion from HepG2 cells. Moreover, RA directly inhibited endothelial cell growth by regulating the expressions of AKT, mTOR, and MAPK pathway related proteins (Figure 7F; Supplementary Figures 3A–C).

### RA Prevents HCC Growth and Progression by Regulating the Expressions of AKT/mTOR and MAPK Pathway Molecules

**Figure 8:**
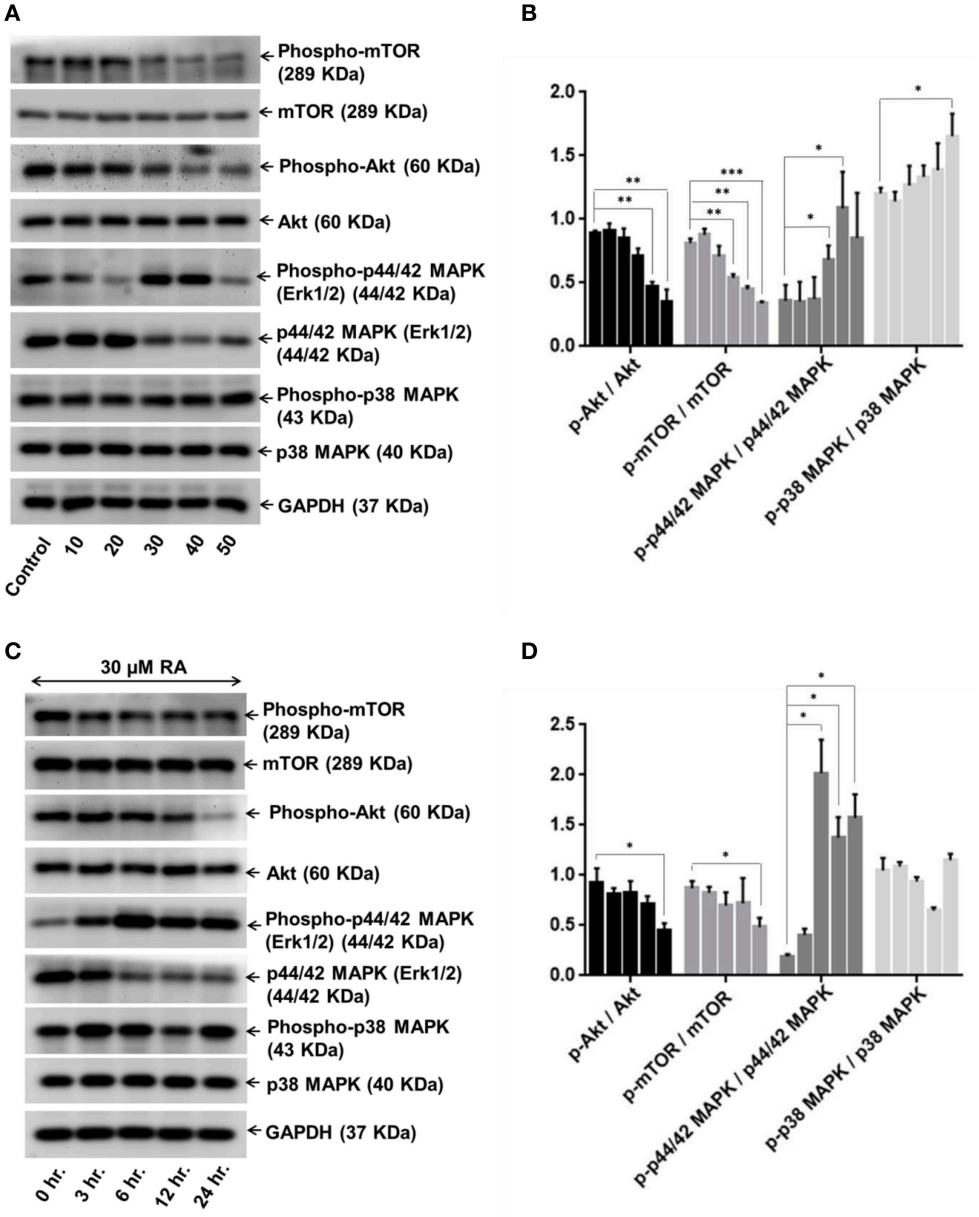
RA modulates AKT/mTOR and MAPK signaling pathways. Representative immunoblots showing the expression levels of AKT/mTOR and MAPK pathway related proteins in RA-treated HepG2 cells in an **(A)** concentration-dependent and **(C)** time-dependent manner. Densitometric analysis of AKT/mTOR and MAPK pathway related proteins in RA-treated HepG2 cells in an **(B)** concentration-dependent and **(D)** time-dependent manner (*n* = 3; ***p ≤ 0.001, **p ≤ 0.01 and *p ≤ 0.05 vs. control/0 hr., respectively).

In order to dissect the mechanisms underlying the inhibitory effects of rotundic acid on the growth and proliferation of hepatocellular carcinoma, we performed western blot experiments with RA treated HepG2 cell lysates to determine the expression of the proteins involved in PI3K/AKT/mTOR and MAPK pathways. A concentration-dependent reduction in the expression levels of phospho-AKT and phospho-mTOR were observed upon RA treatment (Figures 8A,B). The levels of the non-phosphorylated forms of the above proteins remained constant throughout. Conversely, an increase in the levels of phospho-p44/42 MAPK and phospho-p38 MAPK was observed under the same conditions. The expression levels of phospho-p38 MAPK increased in a dose-dependent manner after 30 μM RA treatment, whereas the expression of phospho-p44/42 MAPK increased from 20 to 40 μM RA treatment and then again regressed at 50 μM RA (Figures 8A,B). The expressions of p38 MAPK remained constant throughout whereas the expressions of p44/42 MAPK decreased wherever there was enhanced phospho-p44/42 MAPK expression.

Keeping the treatment concentration of RA at 30 μM, we also performed a time-dependent study to determine the effects of RA treatment on the expressions of PI3K/AKT/mTOR and MAPK pathway molecules. A clear cut time-dependent down-regulation of phospho-AKT was observed upon RA treatment (Figure 8C). Although the expression levels of phospho-mTOR were lower in the 6, 12, and 24 h cell lysate samples as compared to the 0 and 3 h samples, they were not significant when compared to each other (Figure 8C). A significant decrease in the phospho-AKT/AKT and phospho-mTOR/mTOR ratio *w.r.t* the control was only observed after 24 h (Figure 8D). The AKT and mTOR expression levels were constant throughout. We also found that the expression levels of phospho-p44/42 MAPK were higher in the RA treated HepG2 cell lysates obtained at 3 to 24 h time points as compared to the 0 h lysate. The highest expression of phospho-p44/42 MAPK was observed after 6 h of RA treatment. The phospho-p44/42 MAPK expression levels of 12 and 24 h samples were >3 h sample but <6 h sample lysate (Figures 8C,D). Similar to the dose-dependent study, the expression levels of p44/42 MAPK decreased whenever there was an increase in phospho-p44/42 MAPK expression. Greater expressions of phospho-p38 MAPK *w.r.t* control were observed at 3, 6, and 24 h after RA treatment (Figure 8C). A sudden decrease in phospho-p38 MAPK expression was observed after 12 h. For both dose and time-dependent studies, GAPDH was used as the loading control. Our results indicate that RA inhibited angiogenesis and induced apoptosis in HepG2 cells by modulating the expressions of PI3K/AKT/mTOR and MAPK pathway molecules.

### RA Suppresses *in vivo* Tumor Growth in Balb/c Nude Mice

**Figure 9:**
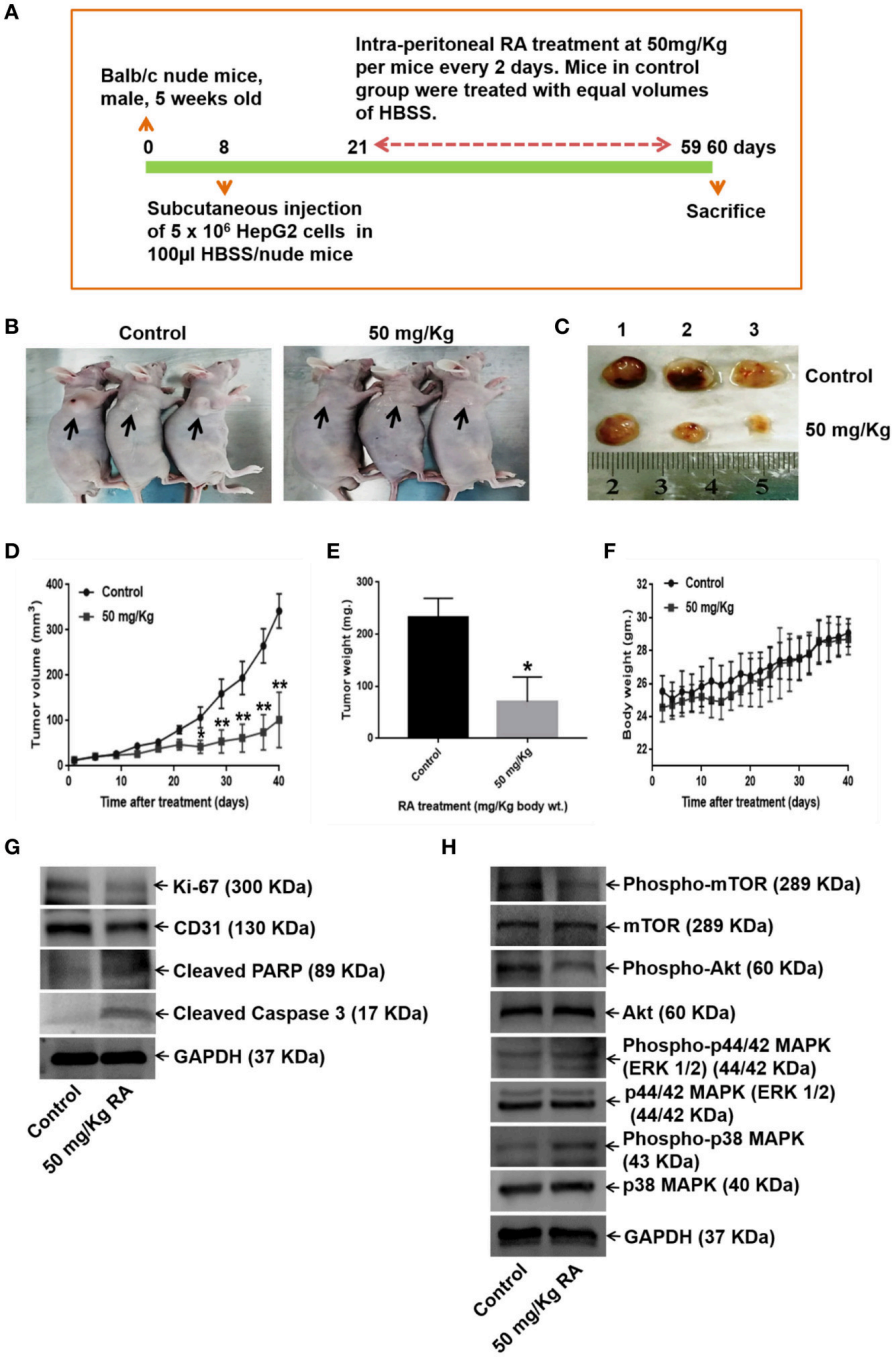
RA abrogates *in vivo* tumor formation in Balb/c nude mice. **(A)** Schematic representation of the experimental protocol. **(B)** Tumor-bearing control and RA-treated mice. **(C)** Mice were euthanized after 60 days; tumors were extracted, weighed, and photographed. Significant reduction of **(D)** tumor volumes and **(E)** tumor weights were observed in the RA-treated group vs. the control group. **(F)** RA did not yield any adverse effects on the mice body weights (**p ≤ 0.01, *p ≤ 0.05). Western blot of tumor tissue lysates indicated that RA. **(G)** attenuated the expressions of Ki-67, CD-31, and induced apoptosis in tumor cells. **(H)** RA modulated the AKT/mTOR and MAPK pathways *in vivo* (*n* = 3).

We further examined the effects of RA on HepG2 tumor-bearing hepatocellular carcinoma mouse model. The data revealed that the intraperitoneal administrations of 50 mg/Kg RA every alternate day attenuated HepG2 tumor weights and tumor volumes significantly as compared to Hank's balanced salt solution (HBSS) treated control mice (Figures 9A–F). On day 60, the mean tumor volumes for the control and the treatment groups were 323.64 and 78.95 mm^3^, respectively; and the tumor growth inhibition (TGI) for the treatment group was found to be 75.6%. Intraperitoneal injections of RA were well-tolerated by the tumor-bearing mice without any significant weight loss.

WB of the tumor tissue lysates demonstrated that RA treatment induced apoptosis (Supplementary Figure S4A), inhibited the expression levels of the proliferation marker Ki-67, angiogenesis marker CD-31 (Supplementary Figure S4A), and AKT/mTOR pathway proteins (Figures 9G,H; Supplementary Figures S4B–G). Although RA notably enhanced he expression levels of phospho-p38 MAPK, significant changes in the expression levels of the phospho-p44/42 MAPK (ERK 1/2) was not observed at the given dose (Figure 9H, Supplementary Figures S4H–I). These results further supported the findings that RA inhibited HCC growth and progression *in vitro*.

## Discussion

Over the last few decades, traditional medicine and natural products have garnered enormous importance as lead compounds for drug discovery (32). Even today, a majority of the world's population relies upon medicinal plants or natural product preparations from plants (herbal extracts) for its primary pharmaceutical care (33–35). Triterpenoids represent the largest group of phytochemicals present in nature and an increasing number of these highly multifunctional molecules have been reported to exhibit anticancer properties *in vitro* as well as in preclinical animal models of cancer (36). Rotundic acid is a pentacyclic triterpenoid obtained from the barks of *Ilex rotunda* and has been testified to possess anticancer properties against several types of cancers. RA and its derivatives have shown to induce apoptosis in cervical cancer and hepatoma cells by mitochondrial degradation and caspase-3 activation (20). In this study, the inhibitory effects of RA on HCC have been highlighted. The results suggested that RA could restrict the cell viability and proliferation of HepG2 and SMMC-7721 cells in a time and dose-dependent manner. RA successfully inhibited the migration and invasion of HCC cells. Moreover, RA also abrogated the extracellular matrix independent cell growth and MMP secretions from HCC cells.

The PI3K/AKT/mTOR pathway is a pro-survival pathway promoting cell growth, metabolism, and angiogenesis in response to extracellular signals. The PI3K/AKT/mTOR pathway regulates many cellular processes, including cell proliferation, survival, growth, and motility that are critically altered during tumorigenesis. Inhibition of this pro-survival pathway molecules has resulted in survival benefits in cancer patients (37–40). The PI3K/AKT/mTOR pathway activation is also associated with drug resistance and has hence evolved as an exciting target for anticancer drug development (41, 42). Activation of the AKT signaling is considered to be a crucial event in human hepatocarcinogenesis. AKT phosphorylation at S-473 has been consistently associated with the poor prognosis and early recurrence of hepatic tumors (43). Once activated, the AKT molecule can directly inhibit apoptosis by phosphorylating the pro-apoptotic proteins such as Bad, Bim, etc., and by controlling the release of cytochrome c from mitochondria, or else, AKT signaling could indirectly stimulate the transcription of the pro-survival genes via IKK phosphorylation and NF-kB activation. AKT also regulates cell survival by activating its downstream substrate, the mTOR, a master regulator of protein translation. Similar to AKT phosphorylation, mTOR activation is also closely associated with the initiation and progression of liver cancers. Activated mTOR signaling has been reported to play a critical role in the malignant transition of hepatocytes to HCC (44). In addition to tumor suppression via apoptosis, the mTOR signaling blockade is also associated with enhanced autophagy in HCC cells (45). Studies have revealed that mTOR/AKT pathways are frequently up-regulated in 40–50% of hepatocellular carcinoma's (46–48). Inhibiting AKT and mTOR signaling have proven beneficial in preventing HCC progression not only by abrogating VEGF secretion but also by modulating the expression of other angiogenic factors such as nitric oxide and angiopoietins (49, 50). Our results show that RA not only induces apoptosis in HepG2 cells by caspase-3 activation/PARP cleavage but also inhibits angiogenesis by suppressing AKT/mTOR signaling pathway molecules in a concentration and time-dependent manner. The therapeutic effects of RA, in part, may be due to its ability to regulate angiogenesis via the AKT/mTOR pathway.

Likewise, aberrant mitogen-activated protein kinase (MAPK) signaling has been reported to play a crucial role in determining response to various treatments in addition to the important part played in cancer development and progression (51). Abnormal p44/42 MAPK (ERK1/2) activity has now been linked to one-third of all human cancers, making it a valuable therapeutic target. The p44/42 MAPK (ERK1/2) acts as a double-edged sword by playing roles in both cell survival and apoptosis. Previous studies suggest that ERK can, both, promote and antagonize apoptosis by regulating the expressions of Bcl 2 family proteins in a case-specific manner (52–55). Similar to the ERK1/2 pathway, dysregulation of the p38 MAPK levels has also been reported in a variety of malignancies, including hepatocellular carcinoma, breast cancer, bladder cancer etc. Although the p38 MAPK acts as a regulator of cell death, it can also mediate cell survival depending upon the type of stimulus in a cell type-specific manner. The selectivity of the p38 MAPK signaling pathway in tumor growth or suppression is unclear (56). Activated p44/42 MAPK (ERK1/2) and its downstream effectors like p38 MAPK have been implicated as critical mediators of several anti-cancer agents including doxorubicin, tamoxifen, and cisplatin (57, 58). As reported earlier, MAPK1/2 and p38 phosphorylation induced cell death and apoptosis via mitochondrial dysfunctioning and caspase activation (59–62). The p38 MAPK also acted as a negative regulator during angiogenesis and played an important role in inhibiting cell migration and invasion by downregulating MMP-2/MMP-9 secretion in cancer cells (63, 64). As shown in this study, RA augmented mitogen-activated protein kinase cascade activation, which could be responsible for the suppressive effects of RA on HCC cell proliferation.

The AKT/mTOR and MAPK signaling pathways have been widely implicated in carcinogenesis and are targeted by numerous FDA approved chemotherapeutic agents for cancer remission and improved survival. Our results are consistent with the previous reports of chemotherapeutic agents providing survival benefits in cancers by modulating AKT/mTOR and MAPK signaling pathways. Our data suggest that rotundic acid could be used for liver cancer chemotherapies with minimal chemotoxic side effects.

## Conclusion

In conclusion, the current study describes RA as an effective inhibitor of HCC growth and proliferation under *in vitro* and *in vivo* conditions. The inhibitory effects of RA on HCC propagation are associated with its ability to inhibit angiogenesis and induce apoptosis by regulating the expressions of AKT/mTOR and MAPK signaling molecules. Taken as a whole, this work substantiates that RA could be useful in treating aggressive malignant diseases and can be further developed as a chemopreventive agent against human hepatocellular carcinoma.

## Data Availability

The raw data supporting the conclusions of this manuscript will be made available by the authors, without undue reservation, to any qualified researcher.

## Ethics Statement

This study was carried out in accordance with National Institute of Health Guidelines for the Care and Use of Laboratory Animals. The study protocol was approved by the Institutional Animal Care and Use Committee of South China University of Technology (Guangzhou, China, Ref: 20180009).

## Author Contributions

GR and SG designed the experiments. GR performed the experiments, analyzed the data, and drafted the manuscript. HL proofread the format and references. SG and LZ revised the article and LZ approved it for final submission.

### Conflict of Interest Statement

The authors declare that the research was conducted in the absence of any commercial or financial relationships that could be construed as a potential conflict of interest.

## References

[1] Bray F Ferlay J Soerjomataram I Siegel RL Torre LA Jemal A. Global cancer statistics 2018: GLOBOCAN estimates of incidence and mortality worldwide for 36 cancers in 185 countries. CA Cancer J Clin. (2018) 68:394–424. 10.3322/caac.21492 30207593

[2] Greten TF Papendorf F Bleck JS Kirchhoff T Wohlberedt T Kubicka S. Survival rate in patients with hepatocellular carcinoma: a retrospective analysis of 389 patients. Br J Cancer. (2005) 92:1862–8. 10.1038/sj.bjc.6602590 15870713 PMC2361778

[3] Berretta M Rinaldi L Di Benedetto F Lleshi A De Re V Facchini G. Angiogenesis inhibitors for the treatment of hepatocellular carcinoma. Front Pharmacol. (2016) 7:428. 10.3389/fphar.2016.00428 27881963 PMC5101236

[4] Mazzocca A Fransvea E Lavezzari G Antonaci S Giannelli G. Inhibition of transforming growth factor beta receptor I kinase blocks hepatocellular carcinoma growth through neo-angiogenesis regulation. Hepatology. (2009) 50:1140–51. 10.1002/hep.23118 19711426

[5] Keating GM. Sorafenib: a review in hepatocellular carcinoma. Target Oncol. (2017) 12:243–53. 10.1007/s11523-017-0484-7 28299600

[6] Heo YA Syed YY. Regorafenib: a review in hepatocellular carcinoma. Drugs. (2018) 78:951–8. 10.1007/s40265-018-0932-4 29915898

[7] Bruix J Tak WY Gasbarrini A Santoro A Colombo M Lim HY Regorafenib as second-line therapy for intermediate or advanced hepatocellular carcinoma: multicentre, open-label, phase II safety study. Eur J Cancer. (2013) 49:3412–9. 10.1016/j.ejca.2013.05.028 23809766

[8] Frenette CT. The role of regorafenib in hepatocellular carcinoma. Gastroenterol Hepatol. (2017) 13:122–4. 28450818 PMC5402683

[9] Ch'ang HJ. Optimal combination of antiangiogenic therapy for hepatocellular carcinoma. World J Hepatol. (2015) 7:2029–40. 10.4254/wjh.v7.i16.2029 26261692 PMC4528276

[10] Frenette C Gish R. Targeted systemic therapies for hepatocellular carcinoma: clinical perspectives, challenges and implications. World J Gastroenterol. (2012) 18:498–506. 10.3748/wjg.v18.i6.498 22363115 PMC3280394

[11] Newman DJ Cragg GM. Natural products as sources of new drugs from 1981 to 2014. J Nat Prod. (2016) 79:629–61. 10.1021/acs.jnatprod.5b01055 26852623

[12] Ji HF Li XJ Zhang HY. Natural products and drug discovery. Can thousands of years of ancient medical knowledge lead us to new and powerful drug combinations in the fight against cancer and dementia? EMBO Rep. (2009) 10:194–200. 10.1038/embor.2009.12 19229284 PMC2658564

[13] Hu Y Wang S Wu X Zhang J Chen R Chen M. Chinese herbal medicine-derived compounds for cancer therapy: a focus on hepatocellular carcinoma. J Ethnopharmacol. (2013) 149:601–12. 10.1016/j.jep.2013.07.030 23916858

[14] Zhou Y Li Y Zhou T Zheng J Li S Li HB. Dietary natural products for prevention and treatment of liver cancer. Nutrients. (2016) 8:156. 10.3390/nu8030156 26978396 PMC4808884

[15] Fan Z Zhou L Xiong T Zhou J Li Q Tan Q. Antiplatelet aggregation triterpene saponins from the barks of Ilex rotunda. Fitoterapia. (2015) 101:19–26. 10.1016/j.fitote.2014.11.007 25447155

[16] Chinese Pharmacopoeia Commission Pharmacopoeia of the People's Republic of China, Vol. I. Beijing: China Medical Science Press (2010).

[17] Kim YJ Jung EB Lee MS Seo SJ Kim MH Lee MW. Rotundarpene inhibits toll-like receptor 2 activation-induced production of inflammatory mediators in keratinocytes by suppressing the Akt and NF-κB pathways. Int Immunopharmacol. (2014) 18:325–32. 10.1016/j.intimp.2013.12.016 24378401

[18] Yang B Li H Ruan QF Xue YY Cao D Zhou XH. A facile and selective approach to the qualitative and quantitative analysis of triterpenoids and phenylpropanoids by UPLC/Q-TOF-MS/MS for the quality control of Ilex rotunda. J Pharm Biomed Anal. (2018) 157:44–58. 10.1016/j.jpba.2018.05.002 29758469

[19] Hsu YM Hung YC Hu L Lee YJ Yin MC. Anti-diabetic effects of madecassic acid and rotundic acid. Nutrients. (2015) 7:10065–75. 10.3390/nu7125512 26633490 PMC4690064

[20] He YF Nan ML Sun JM Meng ZJ Li W Zhang M. Design, synthesis and cytotoxicity of cell death mechanism of rotundic acid derivatives. Bioorg Med Chem Lett. (2013) 23:2543–7. 10.1016/j.bmcl.2013.03.005 23558236

[21] Chen CH Chen MC Wang JC Tsai AC Chen CS Liou JP. Synergistic interaction between the HDAC inhibitor, MPT0E028, and sorafenib in liver cancer cells *in vitro* and *in vivo*. Clin Cancer Res. (2014) 20:1274–87. 10.1158/1078-0432.CCR-12-3909 24520095 PMC4284945

[22] Bhat UG Pandit B Gartel AL. ARC synergizes with ABT-737 to induce apoptosis in human cancer cells. Mol Cancer Ther. (2010) 9:1688–96. 10.1158/1535-7163.MCT-09-0919 20515947 PMC2902270

[23] Gao CF Xie Q Su YL Koeman J Khoo SK Gustafson M. Proliferation and invasion: plasticity in tumor cells. Proc Natl Acad Sci USA. (2005) 102:10528–33. 10.1073/pnas.0504367102 16024725 PMC1180792

[24] Meng J Liu Y Han J Tan Q Chen S Qiao K. Hsp90β promoted endothelial cell-dependent tumor angiogenesis in hepatocellular carcinoma. Mol Cancer. (2017) 16:72. 10.1186/s12943-017-0640-9 28359326 PMC5374580

[25] Chen YW Paliwal S Draheim K Grossman SR Lewis BC. p19Arf inhibits the invasion of hepatocellular carcinoma cells by binding to C-terminal binding protein. Cancer Res. (2008) 68:476–82. 10.1158/0008-5472.CAN-07-1960 18199542 PMC2376045

[26] Taddei ML Parri M Angelucci A Bianchini F Marconi C Giannoni E. EphA2 induces metastatic growth regulating amoeboid motility and clonogenic potential in prostate carcinoma cells. Mol Cancer Res. (2011) 9:149–60. 10.1158/1541-7786.MCR-10-0298 21205836

[27] Tan XW Xia H Xu JH Cao JG. Induction of apoptosis in human liver carcinoma HepG2 cell line by 5-allyl-7-gen-difluoromethylenechrysin. World J Gastroenterol. (2009) 15:2234–9. 10.3748/wjg.15.2234 19437563 PMC2682238

[28] Li DH Li JY Xue CM Han T Sai CM Wang KB. Antiproliferative dimeric aporphinoid alkaloids from the roots of thalictrum cultratum. J Nat Prod. (2017) 80:2893–904. 10.1021/acs.jnatprod.7b00387 29131616

[29] Zhou C Qian L Ma H Yu X Zhang Y Qu W. Enhancement of amygdalin activated with β-D-glucosidase on HepG2 cells proliferation and apoptosis. Carbohydr Polym. (2012) 90:516–23. 10.1016/j.carbpol.2012.05.073 24751072

[30] DeCicco-Skinner KL Henry GH Cataisson C Tabib T Gwilliam JC Watson NJ. Endothelial cell tube formation assay for the *in vitro* study of angiogenesis. J Vis Exp. (2014) e51312. 10.3791/51312 25225985 PMC4540586

[31] Zhang K Chow PK. The effect of megestrol acetate on growth of HepG2 cells *in vitro* and *in vivo*. Clin Cancer Res. (2004) 10:5226–32. 10.1158/1078-0432.CCR-04-0061 15297426

[32] Li JW Vederas JC Drug discovery and natural products: end of an era or an endless frontier? Science. (2009) 325:161–5. 10.1126/science.1168243 19589993

[33] McChesney JD Venkataraman SK Henri JT Plant natural products: back to the future or into extinction? Phytochemistry. (2007) 68:2015–22. 10.1016/j.phytochem.2007.04.032 17574638

[34] Balunas MJ Kinghorn AD. Drug discovery from medicinal plants. Life Sci. (2005) 78:431–41. 10.1016/j.lfs.2005.09.012 16198377

[35] Butler MS Newman DJ. Mother Nature's gifts to diseases of man: the impact of natural products on anti-infective, anticholestemics and anticancer drug discovery. Prog Drug Res. (2008) 65:3–44. 10.1007/978-3-7643-8117-2_1 18084912

[36] Petronelli A Pannitteri G Testa U. Triterpenoids as new promising anticancer drugs. Anticancer Drugs. (2009) 20:880–92. 10.1097/CAD.0b013e328330fd90 19745720

[37] Sandhöfer N Metzeler KH Rothenberg M Herold T Tiedt S Groiß V. Dual PI3K/mTOR inhibition shows antileukemic activity in MLL-rearranged acute myeloid leukemia. Leukemia. (2015) 29:828–38. 10.1038/leu.2014.305 25322685

[38] Fumarola C Bonelli MA Petronini PG Alfieri RR. Targeting PI3K/AKT/mTOR pathway in non small cell lung cancer. Biochem Pharmacol. (2014) 90:197–207. 10.1016/j.bcp.2014.05.011 24863259

[39] Polivka J Jr. Janku F. Molecular targets for cancer therapy in the PI3K/AKT/mTOR pathway. Pharmacol Ther. (2014) 142:164–75. 10.1016/j.pharmthera.2013.12.004 24333502

[40] Albert S Serova M Dreyer C Sablin MP Faivre S Raymond E. New inhibitors of the mammalian target of rapamycin signaling pathway for cancer. Expert Opin Investig Drugs. (2010) 19:919–30. 10.1517/13543784.2010.499121 20569080

[41] LoRusso PM. Inhibition of the PI3K/AKT/mTOR Pathway in Solid Tumors. J Clin Oncol. (2016) 34:3803–15. 10.1200/JCO.2014.59.0018 27621407 PMC6366304

[42] Burris HA 3rd. Overcoming acquired resistance to anticancer therapy: focus on the PI3K/AKT/mTOR pathway. Cancer Chemother Pharmacol. (2013) 71:829–42. 10.1007/s00280-012-2043-3 23377372

[43] Nakanishi K Sakamoto M Yamasaki S Todo S Hirohashi S. Akt phosphorylation is a risk factor for early disease recurrence and poor prognosis in hepatocellular carcinoma. Cancer. (2005) 103:307–12. 10.1002/cncr.20774 15593087

[44] Calvisi DF Wang C Ho C Ladu S Lee SA Mattu S. Increased lipogenesis, induced by AKT-mTORC1-RPS6 signaling, promotes development of human hepatocellular carcinoma. Gastroenterology. (2011) 140:1071–83. 10.1053/j.gastro.2010.12.006 21147110 PMC3057329

[45] Zhang X Yang H Yue S He G Qu S Zhang Z. The mTOR inhibition in concurrence with ERK1/2 activation is involved in excessive autophagy induced by glycyrrhizin in hepatocellular carcinoma. Cancer Med. (2017) 6:1941–51. 10.1002/cam4.1127 28675698 PMC5548872

[46] Villanueva A Chiang DY Newell P Peix J Thung S Alsinet C. Pivotal role of mTOR signaling in hepatocellular carcinoma. Gastroenterology. (2008) 135:1972–83.e1–11. 10.1053/j.gastro.2008.08.008 18929564 PMC2678688

[47] Sieghart W Fuereder T Schmid K Cejka D Werzowa J Wrba F. Mammalian target of rapamycin pathway activity in hepatocellular carcinomas of patients undergoing liver transplantation. Transplantation. (2007) 83:425–32. 10.1097/01.tp.0000252780.42104.95 17318075

[48] Sahin F Kannangai R Adegbola O Wang J Su G Torbenson M. mTOR and P70 S6 kinase expression in primary liver neoplasms. Clin Cancer Res. (2004) 10:8421–5. 10.1158/1078-0432.CCR-04-0941 15623621

[49] Yi T Cho SG Yi Z Pang X Rodriguez M Wang Y. Thymoquinone inhibits tumor angiogenesis and tumor growth through suppressing AKT and extracellular signal-regulated kinase signaling pathways. Mol Cancer Ther. (2008) 7:1789–96. 10.1158/1535-7163.MCT-08-0124 18644991 PMC2587125

[50] Yang Z Xie H He D Li L. Infiltrating macrophages increase RCC epithelial mesenchymal transition (EMT) and stem cell-like populations via AKT and mTOR signaling. Oncotarget. (2016) 7:44478–91. 10.18632/oncotarget.9873 27283897 PMC5190112

[51] De Luca A Maiello MR D'Alessio A Pergameno M Normanno N. The RAS/RAF/MEK/ERK and the PI3K/AKT signalling pathways: role in cancer pathogenesis and implications for therapeutic approaches. Expert Opin Ther Targets. (2012) 16 (Suppl. 2):S17–27. 10.1517/14728222.2011.639361 22443084

[52] Persons DL Yazlovitskaya EM Pelling JC. Effect of extracellular signal-regulated kinase on p53 accumulation in response to cisplatin. J Biol Chem. (2000) 275:35778–85. 10.1074/jbc.M004267200 10958792

[53] Kim YK Kim HJ Kwon CH Kim JH Woo JS Jung JS Role of ERK activation in *cis* platin-induced apoptosis in OK renal epithelial cells. J Appl Toxicol. (2005) 25:374–82. 10.1002/jat.1081 16013042

[54] Ewings KE Hadfield-Moorhouse K Wiggins CM Wickenden JA Balmanno K Gilley R. ERK1/2-dependent phosphorylation of BimEL promotes its rapid dissociation from Mcl-1 and Bcl-xL. EMBO J. (2007) 26:2856–67. 10.1038/sj.emboj.7601723 17525735 PMC1894764

[55] Luciano F Jacquel A Colosetti P Herrant M Cagnol S Pages G. Phosphorylation of Bim-EL by Erk1/2 on serine 69 promotes its degradation via the proteasome pathway and regulates its proapoptotic function. Oncogene. (2003) 22:6785–93. 10.1038/sj.onc.1206792 14555991

[56] Koul HK Pal M Koul S. Role of p38 MAP kinase signal transduction in solid tumors. Genes Cancer. (2013) 4:342–59. 10.1177/1947601913507951 24349632 PMC3863344

[57] Mandlekar S Kong AN. Mechanisms of tamoxifen-induced apoptosis. Apoptosis. (2001) 6:469–77. 10.1023/A:1012437607881 11595837

[58] Olson JM Hallahan AR. p38 MAP kinase: a convergence point in cancer therapy. Trends Mol Med. (2004) 10:125–9. 10.1016/j.molmed.2004.01.007 15102355

[59] Xing W Chen DT Pan JH Chen YH Yan Y Li Q. Lidocaine induces apoptosis and suppresses tumor growth in human hepatocellular carcinoma cells *in vitro* and in a xenograft model *in vivo*. Anesthesiology. (2017) 126:868–81. 10.1097/ALN.0000000000001528 28121635

[60] Cagnol S Chambard JC. ERK and cell death: mechanisms of ERK-induced cell death–apoptosis, autophagy and senescence. FEBS J. (2010) 277:2–21. 10.1111/j.1742-4658.2009.07366.x 19843174

[61] El Mchichi B Hadji A Vazquez A Leca G. p38 MAPK and MSK1 mediate caspase-8 activation in manganese-induced mitochondria-dependent cell death. Cell Death Differ. (2007) 14:1826–36. 10.1038/sj.cdd.4402187 17585337

[62] Taylor CA Zheng Q Liu Z Thompson JE. Role of p38 and JNK MAPK signalling pathways and tumor suppressor p53 on induction of apoptosis in response to Ad-eIF5A1 in A549 lung cancer cells. Mol Cancer. (2013) 12:35. 10.1186/1476-4598-12-35 23638878 PMC3660295

[63] Issbrücker K Marti HH Hippenstiel S Springmann G Voswinckel R Gaumann A. p38 MAP kinase–a molecular switch between VEGF-induced angiogenesis and vascular hyperpermeability. FASEB J. (2003) 17:262–4. 10.1096/fj.02-0329fje 12490545

[64] Chen YJ Cheng YJ Hung AC Wu YC Hou MF Tyan YC. The synthetic flavonoid WYC02-9 inhibits cervical cancer cell migration/invasion and angiogenesis via MAPK14 signaling. Gynecol Oncol. (2013) 131:734–43. 10.1016/j.ygyno.2013.10.012 24145114

