# Supplementary material for: Rotundic Acid Induces DNA Damage and Cell Death in Hepatocellular Carcinoma Through AKT/mTOR and MAPK Pathways
Source: Front Oncol. 2019 Jun 26;9:545. doi: 10.3389/fonc.2019.00545 (PMC6606729; doi:10.3389/fonc.2019.00545)
Supplement: Supplementary file 1 [file Presentation_1.pdf]

Supplementary information

Rotundic acid induces DNA damage and cell death in hepatocellular carcinoma through AKT/mTOR and MAPK pathways.

Gaurab Roy, Su Guan, Hexiang Liu and Lei Zhang

S1. Effect of RA on MMP secretion from SMMC-7721 cells

The effect of RA on the secretion of matrix metalloproteinases was detected by zymography. At the given concentration range (10 µM – 50 µM), RA did not produce substantial effect on the secretion of MMP-2 and MMP-9 from SMMC-7721 cells. However, it is worth mentioning that a substantial reduction was observed at higher concentrations of 60 µM and 80 µM, respectively (Supplementary Figure S1).

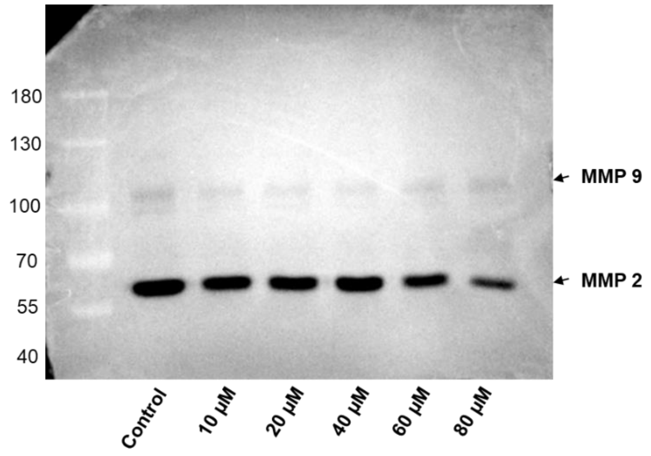

Supplementary Figure S1. RA inhibits MMP secretion in SMMC-7721 cells. Zymography revealed that RA produced slight reduction of MMP-2 and MMP-9 secretion till 50 µM treatment. Substantial reductions were obtained at higher concentrations.

S2. RA induces cell cycle arrest and apoptosis in HCC cells

Flow-cytometry analysis of RA treated HCC cells indicate that RA induces S-phase cell cycle arrest (Supplementary Figure S2A) and apoptosis (Supplementary Figures S2B, S2C) in HCC

cells. Further, apoptosis was also confirmed by DNA ladder assay as mentioned below. Cell lysates of RA treated HepG2 cells were treated with a 1:1 mixture of chloroform: isoamyl alcohol (gentle agitation for 5 min followed by centrifugation). The transparent layers at the top were collected and precipitated in two equivalence of ice-cold ethanol and one-tenth equivalence of sodium acetate. The pellets were then washed twice with 70% ethanol, bench-dried and re-suspended in TE buffer. DNA concentrations were measured using Nanodrop 2000 (Thermo Scientific, USA). The results indicated that RA treatment caused DNA fragmentation in HepG2 cells and the nucleosomal fragments were observed in the form of DNA ladders when run on 1.5% agarose gel (Supplementary Figure S2D).

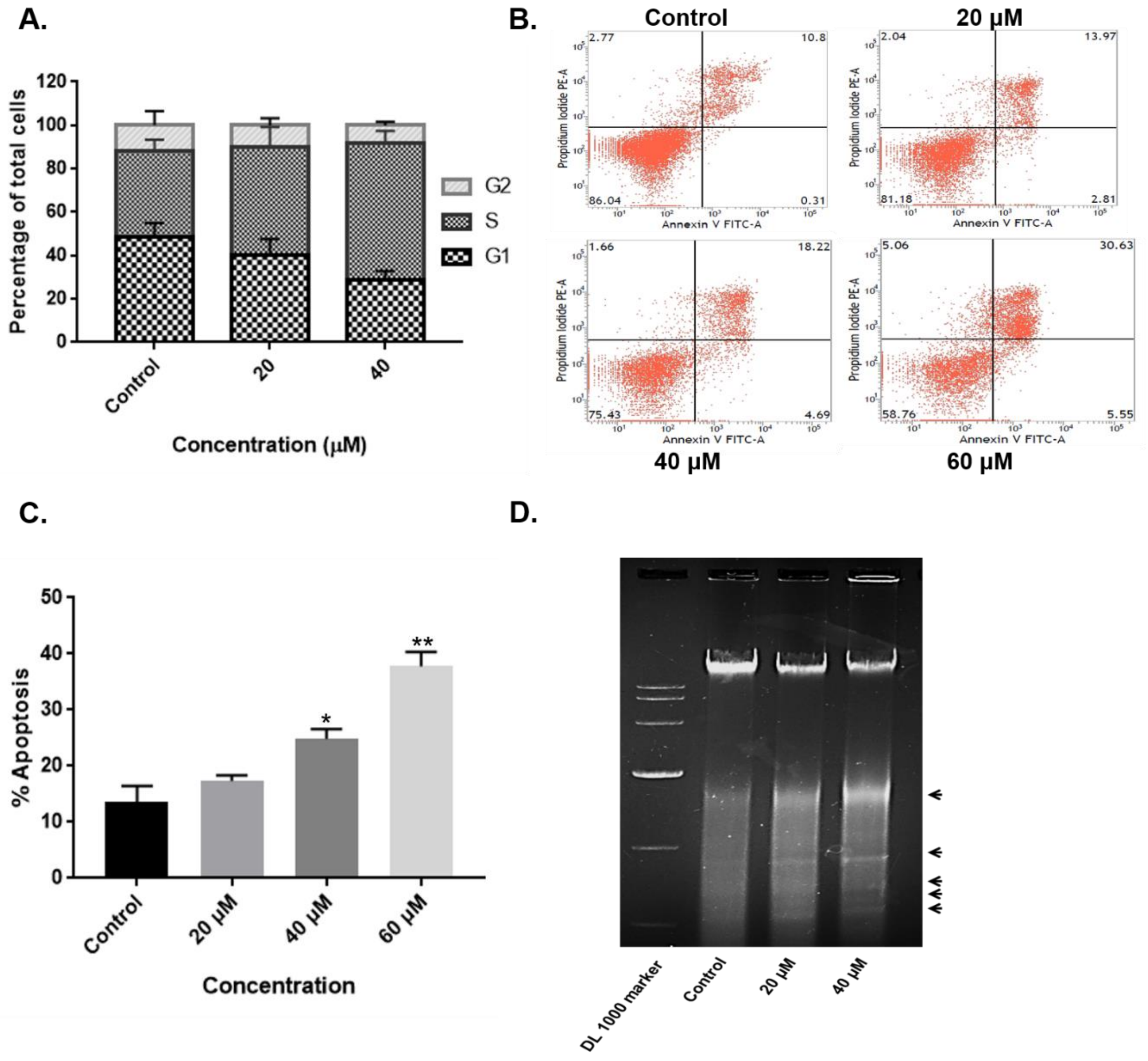

Supplementary Figure S2. **RA induces apoptosis in HCC cells**. (A) RA treatment produced increased accumulation of HepG2 cells in the S-phase of cell cycle. (B, C) RA induced apoptosis in SMMC-7721 cells. (D) Fragmented DNA was observed in the form of DNA ladders when DNA extracted from RA treated HepG2 cells was run on agarose gel. Arrows indicate the small nucleosomal fragments produced as a result of RA-induced apoptosis in HepG2 cells (n = 3, **p ≤ 0.01 and *p ≤ 0.05 *vs.* control).

S3. RA modulates the expression of AKT/ mTOR and p44/42 MAPK proteins in HUVEC`s

Densitometric analysis of the WB results of RA treated HUVEC`s revealed that RA significantly altered the ratio of phospho-AKT/AKT and phospho-p44/42 MAPK / p44/42 MAPK in a concentration-dependent manner. Although RA also inhibited the expression of phospho-mTOR, statistical analysis did not indicate any significant change in the phospho-mTOR/mTOR ratio (Supplementary Figures S3A-C).

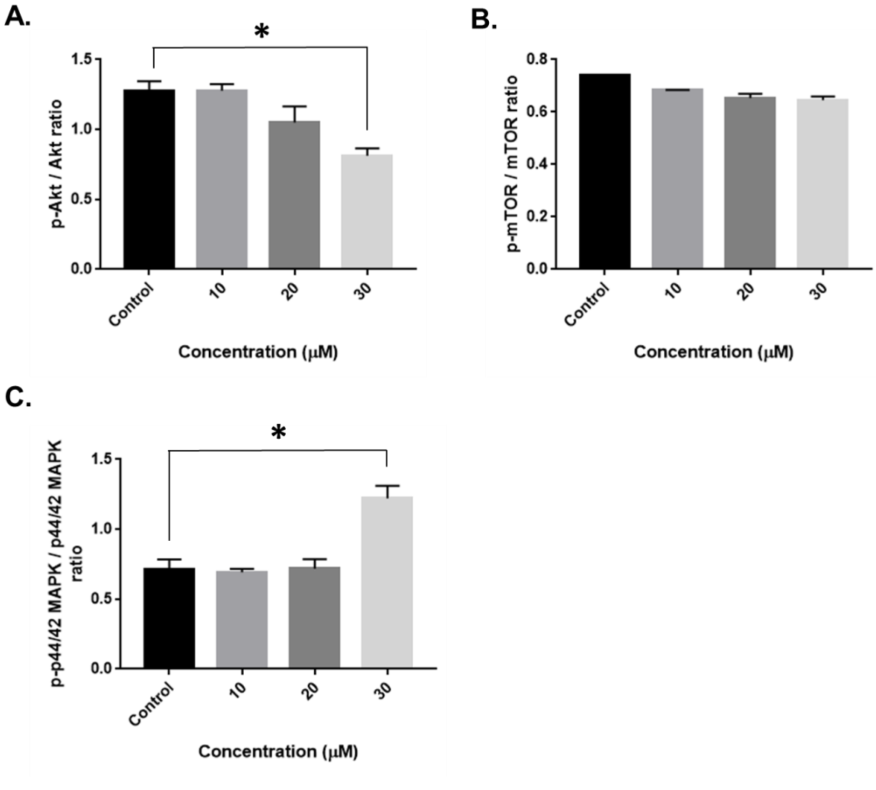

**Supplementary Figure S3. Densitometric analysis of Akt/mTOR and MAPK pathway related proteins in RA treated HUVEC`s**. HUVEC`s were treated with RA and checked for the expression levels of (A) AKT, (B) mTOR, and (C) p44/42 MAPK proteins. RA

significantly altered the expressions of phospho-AKT and phospho-p44/42 MAPK (n = 3 and *$p \leq 0.05$ *vs.* control)

S4. RA inhibited angiogenesis and induced apoptosis in HCC xenograft mouse model by modulating the AKT/mTOR and MAPK pathways

CD-31 staining of the tissue sections were carried out as mentioned below. Briefly, 5-10 µm thick cryostat sections were mounted on gelatin-coated histological slides followed by fixing the samples in formaldehyde fixative solution (85 mM Na_2HPO_4, 75 mM KH_2P0_4, 4% paraformaldehyde) for 20 min at -20 °C. The slides were then rehydrated in PBS for 10 min and incubated in 3% H_2O_2 in water for another 10 min. This was followed by blocking the tissue sections using 2% BSA in PBS for 30 min at room temperature. The slides were then incubated with primary antibody (1:100) overnight at 4 °C in 1% BSA and 0.3% Triton X-100 in PBS. The slides were washed thrice with PBS and incubated with secondary antibody for another 30 min at room temperature. The tissue sections were then covered with DAB (Servicebio, Wuhan, China) for 10 min. The slides were then washed and counter stained with haematoxylin for 5 min and mounted using resin mounting medium. Images were taken at 20X magnification using Pannoramic 250 Flash III (3DHISTECH Ltd., Budapest, Hungary). Results depict that intra-peritoneal administration of RA could suppress angiogenesis in HepG2 xenograft mice (Supplementary Figure S4A).

TUNEL staining of the cryostat sections were also carried out to detect apoptosis. The frozen sections were fixed in cold acetone for 10 min and washed thrice with PBS (pH 7.4). The tissue sections were covered with Proteinase K (Servicebio, China) and incubated for 25 min at 37 °C. Slides were washed and labelled with TUNEL cocktail (Roche, USA) at 37 °C for the next 2 hr in a humidified chamber in dark. The endogenous peroxidases were blocked using H_2O_2. The slides were washed and the tissue sections were further incubated in converter-POD for 30 min at 37 °C. The sections were then stained with DAB as mentioned previously. The slides were washed and the cell nucleus were dyed using sumu differentiation fluid (Servicebio, China). The slices were washed in tap water and dehydrated using 75% ethanol, 85% ethanol, and anhydrous ethanol for 6 min each. The slides were then cleared in xylene for 5 min and mounted using resin mounting medium. Images were taken at 20X magnification using Pannoramic 250 Flash III (3DHISTECH Ltd., Budapest, Hungary). An increased concentration of yellowish-brown colour was seen in the tissue sections sliced out

from the RA treated xenograft mice. The results indicate that RA induces apoptosis mediated HCC cell death in HepG2 xenograft mice (Supplementary Figure S4A).

Moreover, WB of the tumor tissue lysates also supported the *in vitro* findings that RA attenuated HCC cell proliferation, inhibited angiogenesis, and triggered apoptosis by regulating the AKT/mTOR and MAPK pathway molecules (Supplementary Figures S4B-I)

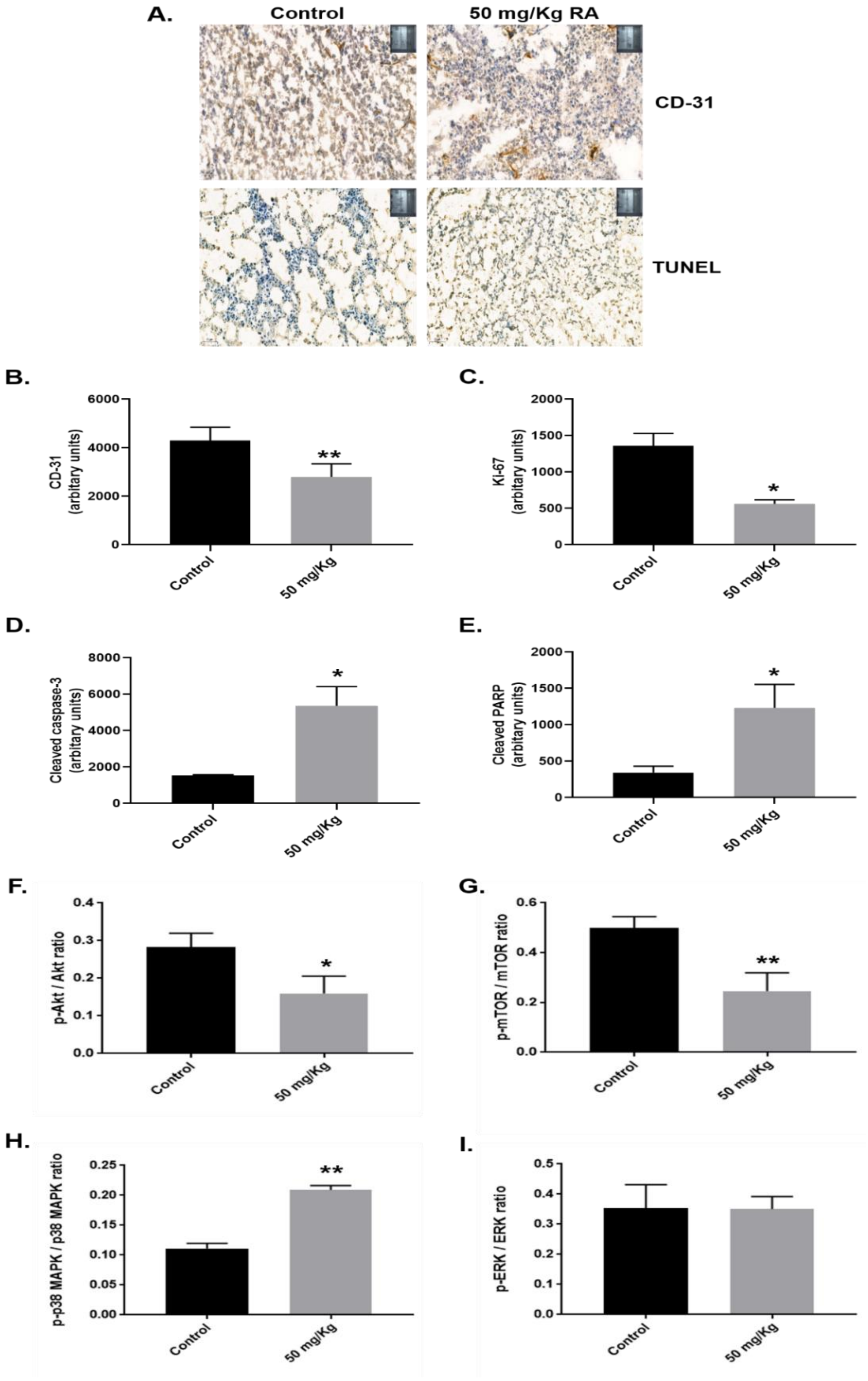

Supplementary Figure S4. Anticancer effects of RA in xenograft mice. (A) CD-31 and TUNEL staining of tumor tissue sections indicated that RA attenuated angiogenesis and induced apoptosis in xenografted tumor bearing mice. (B-I) Densitometric analysis of WB results showed that RA induced apoptosis and altered the expressions of CD-31, Ki-67, AKT/mTOR, and MAPK pathway related proteins in HepG2 xenografted mice (n = 3, $^{**}p \leq 0.01$ and $^{*}p \leq 0.05$ *vs.* control)
